# Physical injuries and burns among refugees in Lebanon: implications for programs and policies

**DOI:** 10.1186/s13031-023-00539-4

**Published:** 2023-09-25

**Authors:** Samar Al-Hajj, Moustafa Moustafa, Majed El Hechi, Mohamad A. Chahrour, Ali A. Nasrallah, Haytham Kaafarani

**Affiliations:** 1 Faculty of Health Sciences, American University of Beirut, Van Dyck Hall, Riad El-Solh, PO Box 11-0236, Beirut, 1107 2020 Lebanon; 2 University of Virginia, Richmond, USA; 3 Division of Trauma, Emergency Surgery and Surgical Critical Care, Massachusetts General Hospital, Harvard Medical School, Boston, MA USA; 4 Department of Surgery, American University of Beirut Medical Center, Beirut, Lebanon; 5 Division of Urology, Department of Surgery, University of Texas Medical Branch, Galveston, TX USA

**Keywords:** Refugee, Injury, Burn, Prevention, Lebanon

## Abstract

**Background:**

Refugees are prone to higher risks of injury due to often austere living conditions, social and economic disadvantages, and limited access to health care services in host countries. This study aims to systematically quantify the prevalence of physical injuries and burns among the refugee community in Western Lebanon and to examine injury characteristics, risk factors, and outcomes.

**Methods:**

We conducted a cluster-based population survey across 21 camps in the Beqaa region of Lebanon from February to April 2019. A modified version of the ‘Surgeons Overseas Assessment of Surgical Need (SOSAS)’ tool (Version 3.0) was administered to the head of the refugee households and documented all injuries sustained by family members over the last 12 months. Descriptive and univariate regression analyses were performed to understand the association between variables.

**Results:**

750 heads of households were surveyed. 112 (14.9%) households sustained injuries in the past 12 months, 39 of which (34.9%) reported disabling injuries that affected their work and daily living. Injuries primarily occurred inside the tent (29.9%). Burns were sustained by at least one household member in 136 (18.1%) households in total. The majority (63.7%) of burns affected children under 5 years and were mainly due to boiling liquid (50%). Significantly more burns were reported in households where caregivers cannot lock children outside the kitchen while cooking (25.6% vs 14.9%, p-value = 0.001). Similarly, households with unemployed heads had significantly more reported burns (19.7% vs. 13.3%, *p* value = 0.05). Nearly 16.1% of the injured refugees were unable to seek health care due to the lack of health insurance coverage and financial liability.

**Conclusions:**

Refugees severely suffer from injuries and burns, causing substantial human and economic repercussions on the affected individuals, their families, and the host healthcare system. Resources should be allocated toward designing safe camps as well as implementing educational awareness campaigns specifically focusing on teaching about heating and cooking safety practices.

**Supplementary Information:**

The online version contains supplementary material available at 10.1186/s13031-023-00539-4.

## Background

Injury is the leading cause of death and disability in people under the age of 44 [1, 2]. While high-income countries (HICs) demonstrated a steady decrease in the rate of injuries over time, low- and middle-income countries (LMICs) showed an increasing trend [3]. The latter is attributed to multiple reasons, particularly the absence of safety regulations in many LMICs and the lack of injury prevention strategies associated with the expanded motorization and the limited resource hospitals and community-based emergency care systems which exacerbate the population’s exposure to numerous hazards and risks of injury [4].

The Eastern Mediterranean Region (EMR) reported the highest rate of injuries related to deaths and disability-adjusted life years (DALYs) among LMICs, particularly injuries related to road traffic crashes and violence [5–8]. According to estimates from the Global Burden of Disease (GBD 2019), injury-related deaths in the EMR were approximately 56.2 per 100,000, ranking the 4th highest rate globally [9]. Wars and regional conflicts have exacerbated injury prevalence in many EMR countries and rendered the provision of healthcare services limited, if not scarce, especially in war-affected regions [10]. The recent Syrian conflict has resulted in what is classified as the worst humanitarian crisis in history [11], with millions of individuals displaced internally or seeking refuge in neighboring countries [12].

Lebanon accommodated the highest number of Syrian refugees relative to its population, estimated at nearly 30% of its current population [13]. The refugee crisis has strained the already fragile Lebanese healthcare system. Several studies have demonstrated a high prevalence of communicable disease [11, 14–16], and injuries of varying aetiologies among the refugee community (e.g. camp burns secondary to open-flame cooking) [17, 18].

Few studies have examined the physical trauma and injury burden sustained by refugee communities, particularly in the EMR countries and Lebanon [16, 19–21]. A regional study evaluated Syrian refugees’ admission to Emergency Department (ED) in Turkey, indicating the overall higher risk of trauma ED admission among refugees compared to the local population, particularly due to head injuries, fractures, dislocations, and sprains of extremities, skin tears and burns [19]. Another study further highlighted the burden of pediatric burns among Syrian refugees in Lebanon, particularly in children aged between 0 and 4 years [16]. A recent local study compared the mechanisms of injuries sustained by refugees compared to residents in Beirut Lebanon and underscored the high prevalence of occupational and violence related injuries among the refugee population [20].

Despite the substantial burden of trauma and injury among the vulnerable refugee population, global health has traditionally focused on communicable diseases with limited attention dedicated to injuries [22]. The Refugee population suffers from a heightened risk of exposure to numerous types of injuries and burns, owing to their substandard living and working conditions [16, 20]. The main objective of this study is to quantify and describe the injury burden among a refugee community in the Northern Beqaa region of Lebanon and offer insights into the injuries' characteristics, extent, risk factors, and outcomes. Understanding the frequency and severity of refugees’ injuries is vital to the planning of injury prevention programs, mitigation of injury impact on the refugee community, and for reducing the strain on the Lebanese healthcare system.

## Methods

### Study setting

The study was conducted in a cluster of refugee camps in the Northwest Beqaa region of Lebanon. Camp sizes ranged from small settlements of 50–100 households, to larger camps which held over 1,000 households. The camp population resided in makeshift tents mostly constructed from cloth, corrugated steel, or wooden planks; thus, lacking adequate cold insulation. Flooring was variable as some households used straw rugs over bare dirt, and others utilized wooden planks. The tents were often crowded, and most households lacked access to electricity or running water.

### Study design and tool adopted

The study was designed as a cross-sectional cluster-based population survey to collect data from 21 refugee camps across the Beqaa Valley in Lebanon, located near the borders with Syria. The Surgeons Overseas Assessment of Surgical Need (SOSAS) tool Version 3.0 (Additional file 1) (www.surgeonsoverseas.org) was adopted in the data collection process. This survey is a validated, cluster-based, cross-sectional tool designed based on the Demographic and Health Surveys (DHS) guidelines and the World Health Organization (WHO) guidelines for conducting community surveys for injuries and violence to determine the burden of surgical conditions within a community [23–27]. Minor modifications were applied to contextualize the tool, tailor it to the Syrian refugee population and expand it to collect specific data on physical injuries and burns. For this study, a burn injury was captured independently and not considered under the umbrella of physical injuries (Additional file 1).

### Data collection

The survey was administered to the head of the refugee household here all injuries sustained by family members over the last 12 months were recorded. The estimated number of refugee population in the region was nearly 4000 residing in camps that the research team were permitted to access. We conveniently sampled households to participate in the study from each camp available households by selecting the 4th residence along the most accessible major routes. If the tent was vacant or absent of adults, the next residence along the route was selected. A total of 750 households participated in the study, accounting for non-responders and refusal to participate households. Participants provided verbal informed consent prior to their participation.

Four bilingual research assistants familiar with the local geography, culture, and Arabic language were recruited and trained to administer the SOSAS questionnaire. The training of data collectors was conducted over a period of two weeks and included simulated practice sessions with role-playing followed by a supervised data collection process at campsites for the initial phases of the study. Data collection took place over three months between February and April 2019.

The study was approved by the American University of Beirut Institutional Review Board (SBS-2018-0561).

### Statistical analysis

The Statistical Package for Social Sciences (SPSS) (version 24) (IBM Corp., Armonk NY, USA) was used for data management and analyses. A descriptive analysis was performed. Continuous data were reported as means with standard deviations, and comparisons were made using the independent t-test. Categorical data were reported as counts with proportions and comparisons reported using the Chi-Square test, or the Fisher's exact test, as appropriate. A two-sided *p*-value < 0.05 was used to indicate statistical significance.

Some questions were unanswered by the study participants, resulting in missing data. Only collected data were analyzed, and no imputation techniques were used for any missing responses. The percentage of missing data for each variable is underlined in the footnote of its respective table.

## Results

### Demographics

A total of 750 heads of households were surveyed. The mean number of individuals per household/informal tented settlement (ITS) was 6.4. The mean age of individuals in each household was 20.4 years. Syrian governorates of origin were diverse with Aleppo (31.7%) and Raqqa (30.0%) being the most highly represented region, followed by Homs (12.2%), Idleb (11.3%), and Hama (7.5%). The average length of stay in Lebanon was 5 years (± 2.5). The majority of refugees were illiterate (59.0%) and nearly (72.2%) were unemployed.

### Prevalence, characteristics, risk factors and outcomes of injuries

A total of 112 households (14.9%) reported sustaining an injury within the past year. Of these, 18 (16.1%) reported being unable to seek healthcare services due to a lack of financial means.

Injuries were sustained at multiple locations with the highest being inside the tent (29.9%) and on the road (28.6%), and less commonly at work fields (i.e., crops and pastures) not within the camp (3.9%) (Table 1). Twenty-two (19.6%) injuries were classified as occupational. Road traffic injuries (RTI) were sustained in 19 households (17%), as mostly (73.7%) were due to a motorcycle crash. None of the injured refugees were adopting any safety measures (e.g., head, or full-face helmets, protective gears, proper boots, etc.) while riding motorcycles.

**Table 1:** Injury distribution in distinct locations

Location	Number (%)
On camp (inside tent)	23 (29.9)
On camp (outside tent)	10 (13)
Road	22 (28.6)
Field	3 (3.9)
Other	19 (24.7)

**Fig. 1:**
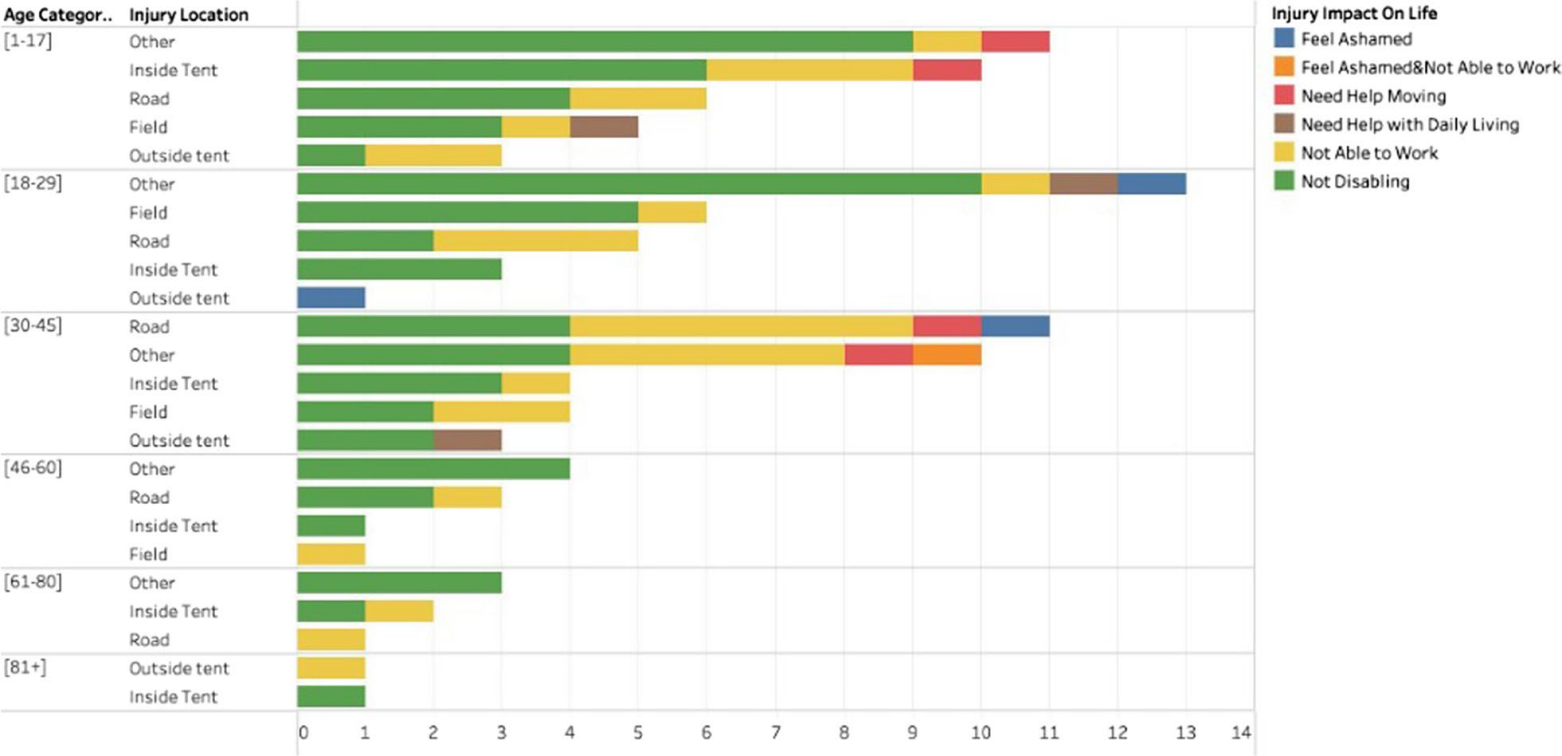
Distribution of injuries by location, age, and impact on life

The effect of injuries varied, with 70 (62.5%) injuries reported as non-disabling, 39 (34.9%) injuries affected individuals’ work and daily living, and 3 (2.7%) injuries were reported as having a psychological impact (i.e., feeling ashamed) (Fig. 1).

### Prevalence, characteristics, and risk factors of burns

A total of 136 households (18.1%) reported a burn to one of the household/tent members. Of those suffering from burns, 67 (53.6%) were males and 58 (46.4%) were females. The mean age of the injured individual was 8.2 years (± 12.4) and the majority (63.7%) of burns were incurred by children less than 5 years. Burns affected different body parts, with the highest rate affecting the hand/arm (50%), leg (19.4%), and face (11.3%) (Table 2).

**Table 2:** Burns distribution on body parts

Body part	Number* (%)
Hand	36 (29)
Face	14 (11.3)
Arm	26 (21)
Leg	24 (19.4)
Foot	13 (10.5)
Multiple	11 (8.9)

*12 unknowns out of 136 total burns

**Fig. 2:**
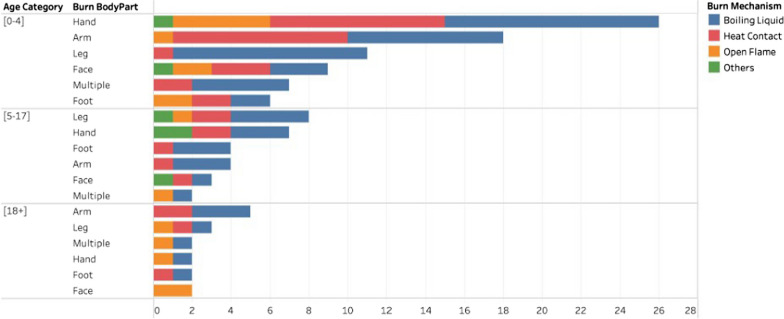
Distribution of burns across age, mechanisms, and body parts

Half of the burns (49.6%) were caused by direct contact with a boiling liquid, 30.9% by contact with hot objects, and 13.8% by contact with an open flame (Fig. 2). The mode and location of cooking varied amongst households. While 78.4% of households use propane for cooking, 21.6% use an open flame. Most cooking occurred inside tents (77%). There was no significant association between the mode or location of cooking and sustaining a burn injury (Table 3). Of the 750 households, 227 (30.3%) reported the inability of child lockout while cooking, i.e., ensuring no child is able to access the cooking area while actively cooking. Those tents had significantly higher reported burn cases (25.6% vs. 14.9%, *p* = 0.001). Households with an unemployed head member were more likely to sustain more burns (19.7% vs. 13.3%, *p* value = 0.05), compared to employed households (Table 3).

**Table 3:** Incidence of burns in association with different tent-related variables

Variable		Number (%)		*p* value
		Burn	No burn	
Tents		136	614	
Mode of cooking	Propane	79 (18.2)	356 (81.8)	0.204
	Open flame	28 (23.3)	92 (76.7)	
Location of cooking	Indoor	78 (18.2)	351 (81.8)	0.187
	Outdoor	30 (23.4)	98 (76.6)	
Child locked	Yes	78 (14.9)	445 (85.1)	0.001*
	No	58 (25.6)	169 (74.4)	
Employed household	Yes	24 (13.3)	147 (86.7)	0.05*
	No	112 (19.7)	457 (80.3)	
Literate household	Yes	36 (14.8)	207 (85.2)	0.102
	No	100 (19.7)	407 (80.3)	

Bold refers to the significant association between variables

*195 tents have an unknown mode of cooking

*Child Locked: the ability to ensure that no children may access the cooking area during active cooking

*Significant association of burns with child locked and employed household (*p* value ≤ 0.05)

## Discussion

This study examined the characteristics, risk factors, and outcomes of injuries affecting the refugee community in Lebanon. Refugee vulnerable status increases individuals’ risk of sustaining various types of injuries, particularly those associated with overcrowded and dire living conditions, in addition to the hazardous work environments. This study provides evidence on the prevalence of injuries and burns among refugees, which serves to tailor data-driven injury prevention programs and strategies applicable to the context of refugees. These preventive measures attempt to mitigate the injury burden on the refugee community and reduce the demand for fragile and limited health care and rehabilitation services in host countries [17, 28, 29].

This study aligns with existing literature and confirms the high prevalence of injuries among the refugee populations in Lebanon [19, 30, 31]. A recent local study indicated the high proportion of medical care services provided to adult Syrian refugees suffering from injuries, compared to local residents in Lebanon [19, 30, 31]. Moreover, regional studies reported that injuries were the leading cause of hospitalization among the refugee population compared to host community [32]. This discrepancy in injury rates was confirmed by various studies examining the risk of injuries among refugees globally; a Canadian study reported an increased rate of motor vehicle injuries, poisoning, suffocation, and overall injury-related hospitalization and mortality among individuals seeking asylum or refugees [30]. A comparable study conducted in Denmark revealed high rates of fatal injuries among refugees [33].

Household injuries were mostly reported in this study. Most refugee injuries occurred in tents, further highlighting the refugees’ suboptimal housing conditions, overcrowded households, and adjacently installed tents. Diminished housing conditions and the absence of safety measures in camps represent major contributors to the high prevalence of injuries among refugees [18]. Road Traffic Injury (RTI) accounts for another contributor responsible for the largest portion of the injury burden. A similar estimate of RTI was reported among Afghan refugees in Pakistan [34], particularly with the absence of safety measures (e.g., wearing helmets, and safety gears) adopted by injured individuals.

Limited access to healthcare services hinders refugees’ ability to receive care following an injury. Timely care is critical to the clinical outcomes, severely impacting the disabilities and disfigurements resulting from injuries or burns. Refugees are often provided with limited guidance on how to navigate and benefit from the healthcare system once settled in host countries, making it even more challenging to access available healthcare services [35, 36]. In the case of Lebanon, the healthcare system is fragile, highly privatized, and under-resourced, making it extremely overburdening to provide care to the local population, let alone the large number of refugees [37]. The increasing healthcare needs of refugees further strains the limited healthcare system resources, forcing refugees to pay out of pocket for their own healthcare expenses or borrow money to cover the costs of treatment for their injuries and burns. [38, 39].

Findings from this study confirm the high prevalence of serious injuries leading to varying levels of physical impairment that affect refugees’ daily living activities. Many of the reported injuries result in severe prognoses, which may lead to permanent disabilities. The latter imposes a ripple effect on refugees’ productivity and limits their integration into the workforce and accordingly hinders their financial capabilities, further increasing refugees’ burden on host countries. A study in war-torn Baghdad found that the rates of permanent disabilities following unintentional injuries were as high as 56% [31]. A similar trend was found in the United Kingdom among a population of refugees and migrants with over one-third of head injuries causing persisting lifelong disability [40].

Similar to physical injury, burns represents a substantial health problem among refugees. The rates reported in this study are comparable to other refugee populations: 11% among Afghan refugees in Pakistan, 17% among Syrian refugees in Turkey, and 7% among Syrian refugees in Belgium [19, 34, 41]. Refugees’ parental education levels (e.g., illiteracy), cultural practices (e.g., child supervision, cooking traditions), and housing conditions (e.g. overcrowded, unsafe heating techniques) are common risk factors that remarkably increase the risk of sustaining injuries among refugees [42, 43]. Camps are often used long beyond their temporary design intention leading to structural failures that often compromise safety. Results of this study show that unemployment and the inability to keep children away from cooking areas are associated with a higher prevalence of burns. Notably, the number of burn injuries was higher in households adopting unsafe cooking practices using open flames instead of propane, however, this association was not statistically significant. Similarly, burn case numbers were higher among households where the head of household reported being illiterate. Although these rates of injuries and burns were not statistically significant, they provide insights into the interlinking multiple factors that influence the occurrences and the severity of these types of injuries among refugees, particularly the children population.

Based on the study findings, a series of recommendations can be proposed to help reduce and control refugees’ high prevalence of injuries and burns. First, refugee camps should be designed with high safety standards focused on avoiding injuries and burns (e.g., a larger lot for each tent to reduce family overcrowding and build camps away from major highways to reduce RTI). Second, a special focus should be given to the safe placement of heating and cooking appliances within camps. Tailored training on safe cooking practices should be considered. Third, adequate parental supervision should be given high priority to reduce incidence of child exposure to burns. Many refugees employed parents tend to assign young children care for older siblings. Parents should ensure child lock and assign child designated play area as a critical step towards preventing children from stepping into floor level cooking and boiling pots. Moreover, parents’ education on first aid burn management is fundamental to suggest alternatives to traditional burn remedies that often times exacerbate the burn long term impact [44–46].

Fourth, adequate Occupational Health and Safety (OHS) training should be provided, focusing on industrial and other high-risk work environments. Finally, refugees should be educated on how to access the local healthcare system and informed of methods for obtaining financial support for health-related needs. Further research warrants the understanding of the circumstances surrounding distinct types of injuries among refugees with the aim to implement tailored injury and burn prevention programs that acknowledge refugees’ context and parents’ level of literacy and to assess the effectiveness of such programs in mitigating the prevalence of injury and burn, particularly among the pediatric population.

To our knowledge, this is the first study to quantify physical injuries and burns among Syrian refugees in Lebanon which has the highest Syrian refugee per capita density. The results of our study can be generalizable to other refugee populations in the Middle East and Northern African (MENA) region with comparable cultural practices and living conditions. This study has several notable limitations. First, data is largely self-reported by household members. Recall bias must be considered as the collected information spans over twelve months. Second, data underreporting is considered another possible limitation of this study. The latter, however, might be mitigated by social concerns and fear of the stigma that may lead to underreporting of injury-related disabilities, particularly those affecting women and children.

## Conclusions

Refugees suffer from a high burden of injuries and burns in Lebanon, with substantial human and economic repercussions on families and the host healthcare system. This study provides ground evidence for the design and development of tailored and context sensitive injury prevention programs targeting refugees. Education on safety guidelines and preventive measures should be introduced as standard protocols among the refugee community. Resources should be allocated for safe camp design, with a special focus on heat appliances and cooking methods. Further research is needed to understand the circumstances surrounding distinct types of injuries among refugees and to implement tailored injury and burn prevention programs that acknowledge refugees’ context and parents’ level of literacy and to assess the effectiveness of such programs in mitigating the prevalence of injury and burn, particularly among the pediatric population.

### Supplementary Information

**Additional file 1.** Surgeons OverSeas Assessment of Surgical Need (SOSAS) Version 3.0.

## Data Availability

The datasets generated during and/or analyzed during the current study are available from the corresponding author on reasonable request.

## Abbreviations

DALYs: Disability-adjusted life years
DHS: Demographic and health surveys
EMR: Eastern Mediterranean Region
GBD: Global burden of disease
HICs: High-income countries
ITS: Informal tented settlement
LMICs: Low- and middle-income countries
MENA: Middle East and Northern African
OHS: Occupational health and safety
RTI: Road traffic injuries
SOSAS: Surgeons Overseas Assessment of Surgical Need
SPSS: Statistical Package for Social Sciences
WHO: World Health Organization
