# Supplementary material for: Physical injuries and burns among refugees in Lebanon: implications for programs and policies
Source: Confl Health. 2023 Sep 25;17:42. doi: 10.1186/s13031-023-00539-4 (PMC10518957; doi:10.1186/s13031-023-00539-4)
Supplement: Supplementary file 1 — Additional file 1. Surgeons OverSeas Assessment of Surgical Need (SOSAS) Version 3.0. [file 13031_2023_539_MOESM1_ESM.pdf]

Surgeons OverSeas Assessment of Surgical Need (SOSAS) Version 3.0

Proposed modification to SOSAS 3.0 for a refugee population in Lebanon SOSAS-R

Are you aware of any hospitals that subsidize care for Syrian refugees?
Do you know which hospitals to go to? [List] _______

If you or someone in your family needed surgery, would you consider traveling to Syria to receive care?
How likely are you to return to Syria for medical/surgical care? Very likely, likely, not likely, very unlikely.
How likely are you to return to Syrian for surgical care even if it is dangerous? Very likely, likely, not likely, very unlikely.

In the past 12 months, have you attempted to receive treatment for any surgical problem?
In the past 12 months has anyone you know attempted to receive treatment for any surgical problem?

What kind of places have you sought medical care at in the past 12 months?

Type (number of times)
NGO__
Govt Hospital ___
Private Hospital ___
Expat doctor___
Other ___
Not seek care___

[open-ended] What other options might you consider if someone needed surgery? ______

Are you aware that the UN (through the UNHCR) can partially subsidize surgical care?
Do you know anyone who has received subsidies for surgery from the UNHCR?
Which hospitals would you consider going to in Lebanon?
  Do you know their names?
  Do you know if they are subsidized?

Questions pertaining to sterility--

Do you have access to running water?
In your house, do you have a mud floor/ (areas of exposed floor)?
Do you have access to a pharmacy?
Do you have access to dressing supplies?

In the winter time, how many layers of clothes must you wear while at home to keep warm?

How welcome do you feel in Lebanon? Very welcome, welcome, not welcome, very unwelcome.

Items in bold are the new questions.
Under the bold items the questions to be asked.
[Items in italics are instructions to the interviewer.]

Paragraphs in the boxes are explanations during the interview for the respondent. Read these out loud and be sure that the person understands the explanation.

A. HOUSEHOLD INFORMATION (TAB: Household)

A1. Number of visits: _____ 1 _____ 2 _____ 3 _____ 4 ☐
[Check off each visit to the household, check the box at the end if this is a replaced household]

A2. Village # OR Cluster #: _____________________

A3. Village Type: ____ Rural _____ Urban _____ Slum

A4. Household Code: ______________

A5. Interviewer Name: ______________

Institutional Review Board
American University of Beirut

10 MAY 2021

APPROVED

‘Good morning/evening. My name is _______________. I am helping with a project from the American University in Beirut. We are trying to find out if there are enough doctors in this area, specifically if there are enough surgeons. A surgeon is a medical doctor who cures patients by taking care of wounds and broken bones or cutting out masses. Sometimes surgeons must put you to sleep to do these things, and other times they must only numb the hurt body part. The purpose of this study is to find out more what the problems are and the reasons it may be hard to receive care.<br><br>To find out if there are enough doctors taking care of these problems in this area, we'd like to ask you and some other members of your household some questions. We will ask questions about health, such as whether members of your household have ever had wounds, broken bones, or masses. By asking these questions, we hope that we can help make access to surgery more easy in this area. We won’t be offering medical care right now, but we hope that the information you provide will help create improved services in the future.<br><br>This survey will take about 30 minutes to 1 hour. All of your responses will be confidential. You have the right to not participate in the survey, or to stop during the interview. First I will ask you some questions about the people who live in this house. After that, I will randomly choose two people from your household and ask them more detailed questions about their health. This information is confidential, and nobody will find out what answers you gave other than me and my research team and we will not collect your name or address. We have an information sheet for you, and we will obtain your consent to participate and separate from the household members who will participate. Do you have any questions at this moment?

A6. Informed consent:
Would you like to participate in this survey?
___ Yes
___ No (If no, what is the reason? _____________)
(no time / no willingness / no reason / no seen benefit / other: explain….)

[Without informed consent you cannot proceed. Make sure the person understands the purpose of this survey. If they don’t want to participate, ask why and mark this.]

Institutional Review Board
American University of Beirut

10 MAY 2021

APPROVED

B. LIVING HOUSEHOLD MEMBERS

The following questions will be about your household members. I want to include every household member that normally eats from the same cooking pot, and slept here last night. We will start the information of the oldest household member and finish with the youngest, also babies and neonates and disabled household members need to be listed in order of their age.

[Fill in all the household members' age and sex in the table, ordered by age, the oldest household person first. Also the household members who are disabled or ill should be mentioned. At the end of the list of household members you need to ask specifically for the newborns and babies in the family and collect the information for each of them in individual tabs.]

B0. Number of household members:
In total, how many people live in your household? _____
*[*Age for babies:*
<3 months = 0
3 - < 6 months = 0.25
6 - < 9 months = 0.5
9 - < 12 months = 0.75
12 - < 24 months = 1 etc.]

Tabs	**B1. Age:** (years*)	**B2. Sex:** Male/Female
1		
2		
3		
4		
5		
6		
7		
8		
9		
10		
11		
12		
13		
14		
15		
16		
17		
18		
19		
20		
21		
22		
23		
24		
25		
26		
27		
28		
29		
30		

Step 1. Check if all information is filled in for all the household members, remember the definition: all the persons eating from the same pot.
Step 2. The total number of household members should be the same as the total number ages and sexes you filled in for question B.
Step 3. Check the order of the household members, should be starting with the oldest person ending with the youngest person, make sure you ask for neonates /babies.
Step 4. Make corrections before you go to step 5.
Step 5. Remember the total numbers of living household members. Open your Random Generator on your iPad: fill in for 'Min':1 and for 'Max' : the number of living household members. Press Generate. The first number which appears corresponds with the number of the household member to interview. Repeat the procedure also to assign the person for the second interview.

Tab number of the assigned household member for the first interview: _____
Tab number of the assigned household member for the second interview: _____

Institutional Review Board
American University of Beirut

10 MAY 2021

APPROVED

C. TRANSPORTATION MEANS

The following questions will be about the health facilities availability for you and your household members, and the transportation you are able to provide for them in need of health care.

C1.1. TRANSPORT TO PRIMARY HEALTH FACILITY:
What is the main way for you or your household members to go to a primary health facility?
___ Public transport (bus/taxi)
___ Car
___ Motorcycle
___ Bicycle
___ Animal
___ On foot
___ Carried

[Call the village/city where this type of facility can be found for the person to be able to respond
Primary health facility: Health facility without functioning operating room
Time guideline: one person can walk 3 miles in one hour or 1 mile takes 20 minutes to walk]

C1.2. Travel time to primary health facility:
How long does it take you in total to get to your primary health facility if you don't have to wait for transportation? (hours)
_____

C1.3. Waiting time for transport:
How long do you probably have to wait for transportation to a primary health facility? (hours)
_____

C1.4. Cost for transport:
What does it cost you to provide transportation to a primary health facility for a sick household member?
_____ 0 – 7,500 (up to $5)
_____ 7,500 – 40,000 (up to $25)
_____ 45,000 – 110,000 (up to $75)
_____ 110,000 – 230,000(up to $150)
_____ 230,000 – 750,000 (up to $500)
_____ 750,000 – 1,515,000 (up to $1000)
_____ >1,515,000 (>$1000)

C1.5. Transport money available?
Are you always able to provide these means for transport of a sick household member?
_____ Yes
_____ No
_____ N/A

C2.1. TRANSPORT TO SECONDARY HEALTH FACILITY:
Are you aware of any SECONDARY health facilities? *If no, skip.*
What is the main way for you or your household members to go to a secondary health facility?
___ Public transport (bus/taxi)
___ Car
___ Motorcycle
___ Bicycle
___ Animal
___ On foot
___ Carried

[Call the village/city where this type of facility can be found for the person to be able to respond
Secondary health facility: Health facility with functioning operating room
Time guideline: one person can walk 3 miles in one hour or 1 mile takes 20 minutes to walk]

C2.2. Travel time to secondary health facility:
How long does it take you in total to get to your secondary health facility if you don't have to wait for transportation? (hours)
_____

C2.3. Waiting time for transport:
How long do you probably have to wait for transportation to a secondary health facility? (hours)
_____

Institutional Review Board
American University of Beirut

10 MAY 2021

APPROVED

C2.4. Cost for transport:
What does it cost you to provide transportation to a secondary health facility for a sick household member? (Lebanese Pound or USD)
_____ 0 – 7,500 (up to $5)
_____ 7,500 – 40,000 (up to $25)
_____ 45,000 – 110,000 (up to $75)
_____ 110,000 – 230,000(up to $150)
_____ 230,000 – 750,000 (up to $500)
_____ 750,000 – 1,515,000 (up to $1000)
_____ >1,515,000 (>$1000)

C2.5. Transport money available?
Are you always able to provide these means for transport of a sick household member?
_____ Yes
_____ No
_____ N/A

C3.1. TRANSPORT TO TERTIARY HEALTH FACILITY:
What is the main way for you or your household members to go to your tertiary health facility?
___ Public transport (bus/taxi)
___ Car
___ Motorcycle
___ Bicycle
___ Animal
___ On foot
___ Carried

[Call the village/city where this type of facility can be found for the person to be able to respond
Tertiary health facility: *Health facility with functioning operating room and minimal one surgical specialists (Surgeons/Orthopedics/Gynecologist/Urologist)*
Time guideline: one person can walk 3 miles in one hour or 1 mile takes 20 minutes to walk]

C3.2. Travel time to tertiary health facility:
How long does it take you in total to get to your tertiary health facility if you don't have to wait for transportation? (hours)
_____

C3.3. Waiting time for transport:
How long do you probably have to wait for transportation to a tertiary health facility? (hours)
_____

C3.4. Cost for transport:
What does it cost you to provide transportation to a tertiary health facility for a sick household member? (Leones)
_____ 0 – 7,500 (up to $5)
_____ 7,500 – 40,000 (up to $25)
_____ 45,000 – 110,000 (up to $75)
_____ 110,000 – 230,000(up to $150)
_____ 230,000 – 750,000 (up to $500)
_____ 750,000 – 1,515,000 (up to $1000)
_____ >1,515,000 (>$1000)

C3.5. Transport money available?
Are you always able to provide these means for transport of a sick household member?
_____ Yes
_____ No
_____ N/A

Institutional Review Board
American University of Beirut
10 MAY 2021
APPROVED

D. DECEASED HOUSEHOLD MEMBERS

D0. Number of household deaths:
Did you have any household member who passed away in the past year?
“I'm very sorry to hear that”

_____
[Ask specifically for neonates and babies.]

[FOLLOWING QUESTIONS ONLY IF THERE WAS A DECEASED HOUSEHOLD MEMBER IN THE LAST YEAR, UNDER ROW 1 THE ANSWERS FOR THE FIRST HOUSEHOLD DEATH, CONTINUE WITH THE FOLLOWING ROWS IF THERE WERE MORE HOUSEHOLD DEATHS IN THE LAST YEAR]

‘I'm sorry to hear that you lost a household member in the last year. The following questions are about this/these deceased person(s).’

1…..	2…..	3…..	4…..	5…..

D1. Age at death:
How old was the household member when she/he died?
[Age for babies:
<3 months = 0
3 - <6 months = 0.25
6 - <9 months = 0.5
9 - <12 months = 0.75
12 -<24 months = 1 etc.]

1…..	2…..	3…..	4…..	5…..

D2. Sex:
What was the sex of the household member?

Male
Female

D2.1 Pregnant at death: Yes = 1 No = 2

1…..	2…..	3…..	4…..	5…..

Was the household member pregnant when she died or did she deliver within 6 weeks of her death?

D3.1. Death specifics:

How many people from your household died in the past 3 years?
Did anyone die directly because of the war since 2011?
How many died because they could not get medical care?

Did the household member have any of the following problems less than a week before s/he died?
For neonates / babies: Did the child look normal and could it drink, urinate and defecate normally after birth?

1…..	2…..	3…..	4…..	5…..

[Show empathy for the story told. Don't be judgmental in any way; let the person tell his/her story. For definitions see Question F2.1]

Injury
Wound not due to an injury
Bleeding or ill around childbirth
Mass (Growth or Swelling)
Deformity congenital
Deformity acquired
Abdominal distention or pain
None of the above

Institutional Review Board
American University of Beirut

10 MAY 2021

APPROVED

D3.1.1 Type of injury / accident: [Only if D3.1 is answered with 'Injury']
Did the problem started after an injury or accident? What kind of accident?

1…..	2…..	3…..	4…..	5…..

[Pick the one that best describes the injury / accident. Pedestrian and bicycle crash definition: there was no motorized vehicle involved. All can be intentional or unintentional.]

Car, truck, bus crash
Motorcycle crash
Pedestrian, bicycle crash
Gunshot / firearm
Stab / slash / cut / crush
Bite or animal attack
Fall
Open fire / explosion
Hot liquid / hot object

1…..	2…..	3…..	4…..	5…..

D4. Healthcare sought:
Did the household member go to a health facility or see a doctor/nurse before she/he died?

Yes
No

1…..	2…..	3…..	4…..	5…..

D4.1.1. Traditional Healer:
Did you go to a traditional healer, traditional doctor, or bone setter for this problem?

Yes
No

1…..	2…..	3…..	4…..	5…..

D4.1.2. Expat Healer:
Did you go to a Syrian physician who lives in Lebanon?

No

D5. Type of healthcare received: *[only if D4 is yes]*

1…..	2…..	3…..	4…..	5…..

What kind of treatment did the household member receive?

None / No surgical care
Major procedure = a procedure which requires regional or general anesthesia
Minor procedures = dressings, wound care, punctures, suturing and I&D

D6. Reason for not having surgical care: *[Only if D4 is 'No' or D5 is 'No surgical care']*
[If the person went for traditional medicine ask why and mark that answer]
What was the main reason not to go to a health facility to see a

Institutional Review Board
American University of Beirut

10 MAY 2021

APPROVED

1…..	2…..	3…..	4…..	5…..

doctor/nurse or not to have an operation or dressings?

No money for health care
No (money for) transportation
No time (person died before arrangements)
Fear / no trust
Not available (facility/personnel/equipment)
No need (condition is not surgical)

1…..	2…..	3…..	4…..	5…..

D7. Location of death:

Where did the household member die?

Home
Health Facility
Somewhere else

D8. Give a brief explanation of the story told.

____

____

____

‘Thank you very much for answering these questions. Let me go quickly over the survey to check everything.’

[Go over all the tabs to ensure that you have everything, ask the questions again which you accidentally skipped.]

I checked the household information tab, there is no data missing:
_______________ *[date]* _______________ *[name]* _______________ *[signature of interviewer]*

‘Thank you very much for giving all this information. I have a letter with contact information for you if you would have questions for us about this survey. Can I speak to the household members who I have randomly chosen to ask them more specific questions about their health?’

[Give the letter with the (contact) information about the survey. If the household members are not available now, you should make an appointment for later on that day or the next day.] [TIME: ____ hour ____ min]

IF THERE WERE MORE THAN ONE DECEASED HOUSEHOLD MEMBER, FILL IN THE SECOND OR THIRD PERSONS DETAILS IN THE APPROPRIATE SPACES (THE TABS OR COLUMNS WITH 2 OR 3 RESPECTIVELY.

THE FOLLOWING QUESTIONS IN SECTION E, F, G, H, I, J, K, L , ARE TO BE ASKED TO THE TWO HOUSEHOLD MEMBERS OF THE FAMILY WHO WERE RANDOMLY SELECTED FOR THE INTERVIEW.

Institutional Review Board
American University of Beirut

10 MAY 2021

APPROVED

IN THE IPAD VERSION THIS IS DISPLAYED AS EXTRA TABS, IN THE PAPER FORMAT THIS IMPLIES DOUBLE PRINTING OF THE FOLLOWING QUESTIONS. PERMANENT ATTACHMENT OF THE FILES TOWARDS THE HOUSEHOLD INFORMATION IS NEEDED TO CONNECT THE INDIVIDUAL DATA WITH THE HOUSEHOLD AND CLUSTER DATA.

Institutional Review Board
American University of Beirut

10 MAY 2021

APPROVED

E. GENERAL INFORMATION (Survey 1I)

E1. Household list (ID) number:

_____

FIRST RESPONDENT [TIME: ____ hour ____ min]

[from the table with the questions B1 and B2]

[Check this box if this is a replaced household member] ☐

[If a surrogate is answering for a child, ask all the questions about the child.]

E2. Sex:

___ Male

___ Female

E3. Age:

____

[Age for babies:

<3 months	*= 0*
3- <6 months	*= 0.25*
6- <9 months	*= 0.5*
9- <12 months	*= 0.75*
12-24 months	*= 1 etc.]*

Good morning/evening. My name is _______________. I am working on a project to help understand about medical care in this area.. We are trying to find out if there are enough doctors in this area, specifically if there are enough surgeons. A surgeon is a medical doctor who cures patients by taking care of wounds and broken bones or cutting out masses. Sometimes surgeons must put you to sleep to do these things, and other times they must only numb the hurt body part.

To find out if there are enough doctors taking care of these problems in this area, we'd like to ask you some questions. We will ask you questions about your health, such as whether you have ever had wounds, broken bones, or masses. By asking you these questions, we hope that we can help make more skilled doctors available in your village. We won't be offering medical care right now, but we hope that the information you provide will help create improved services in the future.

This survey will take about 30 minutes. All of your responses will be confidential. You have the right to not participate in the survey, or to stop during the interview. We have an information sheet for you, and we will obtain your consent to participate. Do you have any questions at this moment?

E4. Informed consent: Would you like to participate in this survey? OR
Is it okay for your son/daughter to participate in this survey?

___ Yes

___ No, what is the reason? (no time / no willingness / no reason / no seen benefit / other: explain….)

[For minors (individuals under age 18), this consent E4 is obtained from a guardian/parent. Without informed consent you cannot proceed. Make sure the person understands the purpose of this survey. If they don't want to participate, ask why and mark this.]

E.5 Minor assent: *[under 18 years of age only]* Would you like to participate in this survey?

___ Yes

___ No, what is the reason? (no time / no willingness / no reason / no seen benefit / other: explain….)

___ Surrogate consent only

[Surrogate consent only: this can be used in case of a child under 12 years of age, who is not around for the interview. The guardian/parent can answer all the questions for the child.
When the child is around, the permission to participate (assent) is asked and only with the assent of the child the interview is held together with the guardian/parent. For children over the age of 12 the guardian/parent can be around for the interview depending on the wish of the child.]

The following questions are general questions, later on I will ask more about your health.

E6. Education:

What is the highest educational level that you have achieved or are currently following?

___ None (includes nursery)

___ Primary school

___ Secondary school (junior / senior)

___ Tertiary (diploma, colleges, bachelors)

___ Graduate degree (Master degree, PhD)

E6.1 Literacy:

Are you able to read and write in any language?

[For adults and children who are currently learning how to read and write answer: 'No']

___ Yes

___ No

Institutional Review Board
American University of Beirut

10 MAY 2021

APPROVED

E7. Occupation:
What is your primary occupation?

___ Unemployed	*[Currently looking for jobs, retiree's, students]*
___ Home maker	*[Housewives]*
___ Domestic helpers	*[Cleaners, housekeepers, watch guards]*
___ Farmer	*[Herders, agriculture, pastoralist]*
___ Self-employed / small-business	*[Small business owners like: shops, kiosks, food traders]*
___ Government employee	*[Police officer, accountant, teachers, health care workers]*
___ Non-government employee	*[Cooperation managers, NGO-staff]*

E8. Origin?
What is your ethnic background?
_____ Syrian
_____ Palestinian
_____ Iraqi
_____ Kurdish
_____ Yemeni
_____ Lebanese
_____ Egyptian
_____ Other: ___________

E 8.1 Syrian Origin?
If answered [Yes] to Syrian, where in Syrian are you from?
_____ Damascus
_____ Eastern Ghouta
_____ Western Ghouta
_____ Aleppo
_____ Hama
_____ Deir el Zor
_____ Daraa
_____ Idlib
_____ Latakia
_____ Tartus

E9. Length of stay in house:
How many years have you lived in this household?
_____ *[years]* _______ *[months]*

E9.1. Length of stay in Lebanon:
How many years have you lived in Lebanon?
_____ *[years]* _______ *[months]*

E10. Health status:
Are you generally healthy?
___ Yes
___ No
[if 'Yes' to E10. The following questions E11, E12 and E13, can be skipped]

E11. Time ill:
In total how many weeks have you been ill during the past year?
_____ *[weeks]*

E12. Number of health facility visits:
How many times have you visited a clinic or hospital, or nurse / medical doctor in the last year?
_____

E13. Recovery from illness:
Have you recovered fully from the illness you had?
___ Yes
___ No

EXPLAIN:

Institutional Review Board
American University of Beirut

10 MAY 2021

APPROVED

Surgery, also known as an operation, can be done for a swelling, mass, abdominal pain, and many other things. Patients often have a bandage after having surgery or may need to stay in the hospital for some time. Sometimes, children are born with problems that can be fixed with an operation. Examples of these problems are open lips, missing anus, or strange feet.

Some people who break a bone or have a wound, may not have an operation but still need to be seen by a doctor or stay in the surgical ward of a hospital. Since this does not include an operation, but includes surgical consultation, we call it surgical care.

Now I'm going to ask you about all the surgical problems you've had in your lifetime. We'll start with your head and move all the way down to your toes.

Institutional Review Board
American University of Beirut

10 MAY 2021

APPROVED

F. FACE / HEAD / NECK

F1. Face / head / neck:
Have you ever had a wound, burn, mass / goiter, deformity, problem with eating/drinking, a problem with your eyes or ears or an operation on your face, head, or neck?
___ Yes
___ No

[IF THERE WERE/ARE NO PROBLEMS WITH THIS ANATOMICAL SECTION YOU CAN CONTINUE WITH SECTION G. IF THE PERSON HAD A PROBLEM WITH THIS ANATOMICAL SECTION YOU QUESTION FIRST ON PROBLEM 1 ALL THE QUESTIONS ON THIS PAGE AND GO FURTHER IF HERE ARE MORE PROBLEMS]

F1.1. Face / head / neck location:

Problem 1	Problem 2	Problem 3

On what part of your head / neck / face did the problem occur?

Eye
Ear / nose / throat
Dental / lips / mouth
Neck
Head

F2.1. Face / head / neck specifics:

Problem 1	Problem 2	Problem 3

Tell me what problem you have had.

Wound injury related
Wound not injury related
Burn
Mass or growth / goiter
Deformity congenital
Deformity acquired

[Wound: Open skin; sometimes leaking blood, pus or liquid
Deformity: An abnormal tissue arrangement or malformation
Congenital: The person is born with the problem. Think about: cleft lips, hydrocephalus etc.
Acquired: The person got the problem later in life. Think about: scars and broken bones]

F2.1.1 Type of injury / accident:
Did the problem started after an injury or accident? What kind of accident?

Problem 1	Problem 2	Problem 3

[Pick the one that best describes the injury / accident. Pedestrian and bicycle crash definition: there was no motorized vehicle involved. All can be intentional or unintentional.]

No, it was not due to an injury / accident
Car, truck, bus crash
Motorcycle crash
Pedestrian, bicycle crash
Gunshot
Stab / slash / cut / crush

Institutional Review Board
American University of Beirut
10 MAY 2021
APPROVED

Bite or animal attack
Fall
Open fire / explosion
Hot liquid / hot object

Problem 1	Problem 2	Problem 3

F3.1. Timing:
When did this problem start? In the last month
During the past 12 months but longer than a month ago
Longer than 12 months ago

Problem 1	Problem 2	Problem 3

F3.1.1 At this moment:
Do you have this problem now (or during the last week)? Yes
No

Problem 1	Problem 2	Problem 3

F4.1. Healthcare sought:
Did you go to a health facility or see a doctor/nurse for this problem? Yes No

Problem 1	Problem 2	Problem 3

F4.1.1. Traditional Healer:
Did you go to a traditional healer, traditional doctor, or bone setter for this problem? Yes
No

Institutional Review Board
American University of Beirut

10 MAY 2021

APPROVED

F5.1. Type of healthcare received: *[Only when 'yes' to F4.1]*
What kind of treatment did you receive?

Problem 1	Problem 2	Problem 3

None / No surgical care
Major procedure = a procedure which requires regional/general anesthesia
Minor procedures = dressings, wound care, punctures, suturing and I&D

F6.1. Reason for not having surgical care: *[Only when 'none / no surgical care' to F5.1 or 'no' to F4.1]*
What was the main reason not to go to a health facility to see a doctor/nurse or not to have an operation or dressings?
[If the person went to a traditional healer ask why (s)he didn't go to a health care facility and mark that as the answer. If the person was referred but did not go to the referral hospital, mark the answer why (s)he did not go here.]

Problem 1	Problem 2	Problem 3

No money for health care
No (money for) transportation
No time
Fear / no trust
Not available (facility/personnel/equipment)
No need

F7.1. Disability:

Problem 1	Problem 2	Problem 3

Does this problem still impact your daily life?

The condition is not disabling
I feel ashamed
I'm not able to work like I used to
I need help with transportation
I need help with daily living

[Disability: a physical problem that impacts your life, or makes it difficult to carry out your daily activities.]

[ASK IF THE PERSON HAD ANOTHER PROBLEM REGARDING THIS ANATOMICAL LOCATION. IF SO, GO TO THE FOLLOWING ROW, PROBLEM 2 OR 3, TO REGISTER THIS PROBLEM. IF HERE ARE NO OTHER PROBLEMS REGARDING TO THIS LOCATION YOU CAN GO TO THE FOLLOWING ANATOMICAL LOCATION. IN CASE OF MORE THAN 3 PROBLEMS, MARK THE ONES WHICH ARE MOST PRESENT AND MOST RELEVANT FOR THE RESPONDENT]

G. CHEST / BREAST (TAB: Survey Part II)

G1. Chest / breast:
Have you ever had a wound, burn, breast mass, deformity, or an operation on your chest (including heart or lungs) or breast?
___ Yes
___ No

[IF THERE WERE/ARE NO PROBLEMS WITH THIS ANATOMICAL SECTION YOU CAN CONTINUE WITH THE FOLLOWING. IF THE PERSON HAD A PROBLEM WITH THIS ANATOMICAL SECTION YOU QUESTION FIRST ON PROBLEM 1 ALL THE QUESTIONS ON THIS PAGE AND GO FURTHER IF HERE ARE MORE PROBLEMS]

G2.1. Chest / breast specifics:

Problem 1	Problem 2	Problem 3

Institutional Review Board
American University of Beirut

10 MAY 2021

APPROVED

Tell me what problem you have had.

Wound injury related
Wound not injury related
Burn
Breast mass / breast cancer
Deformity congenital
Deformity acquired

[Wound:	*Open skin; sometimes leaking blood, pus or liquid*
Deformity:	*An abnormal tissue arrangement, malformation*
Congenital:	*The person is born with the problem. Example: heart malformation*
Acquired:	*The person got the problem later in life]*

Institutional Review Board
American University of Beirut

10 MAY 2021

APPROVED

G2.1.1 Type of injury / accident:
Did the problem started after an injury or accident? What kind of accident?

Problem 1	Problem 2	Problem 3

[Pick the one that best describes the injury / accident. Pedestrian and bicycle crash definition: there was no motorized vehicle involved. All can be intentional or unintentional.]

No, it was not due to an injury / accident
Car, truck, bus crash
Motorcycle crash
Pedestrian, bicycle crash
Gunshot
Stab / slash / cut / crush
Bite or animal attack
Fall
Open fire / explosion
Hot liquid / hot object

Problem 1	Problem 2	Problem 3

G3.1. Timing:
When did this problem start?

In the last month
During the past 12 months but longer than a month ago
Longer than 12 months ago

Problem 1	Problem 2	Problem 3

G3.1.1 At this moment:
Do you have this problem now (or during the last week)? Yes
No

Problem 1	Problem 2	Problem 3

G4.1. Healthcare sought:
Did you go to a health facility or see a doctor/nurse for this problem? Yes
No

Problem 1	Problem 2	Problem 3

G4.1.1. Traditional Healer:
Did you go to a traditional healer, traditional doctor, or bone setter for this problem? Yes
No

Problem 1	Problem 2	Problem 3

Institutional Review Board
American University of Beirut

10 MAY 2021

APPROVED

G5.1. Type of healthcare received: *[Only when 'Yes' to G4.1]*
What kind of treatment did you receive?

None / No surgical care
Major procedure = a procedure which requires regional/general anesthesia
Minor procedures = dressings, wound care, punctures, suturing and I&D

G6.1. Reason for not having surgical care: *[Only when 'none / no surgical care' to G5.1 or 'no' to G4.1]*
What was the main reason not to go to a health facility to see a doctor/nurse or not to have an operation or dressings?
[If the person went to a traditional healer ask why (s)he didn't go to a health care facility and mark that as the answer.
If the person was referred but did not go to the referral hospital, mark the answer why (s)he did not go here.]

Problem 1	Problem 2	Problem 3

No money for health care
No (money for) transportation
No time
Fear / no trust
Not available (facility/personnel/equipment)
No need

G7.1. Disability:

Problem 1	Problem 2	Problem 3

Does this problem still impact your daily life?

The condition is not disabling
I feel ashamed
I'm not able to work like I used to
I need help with transportation
I need help with daily living

[Disability: a physical problem that impacts your life, or makes it difficult to carry out your daily activities.]
[ASK IF THE PERSON HAD ANOTHER PROBLEM REGARDING THIS ANATOMICAL LOCATION. IF SO, GO TO THE FOLLOWING ROW, PROBLEM 2 OR 3, TO REGISTER THIS PROBLEM. IF HERE ARE NO OTHER PROBLEMS REGARDING TO THIS LOCATION YOU CAN GO TO THE FOLLOWING ANATOMICAL LOCATION. IN CASE OF MORE THAN 3 PROBLEMS, MARK THE ONES WHICH ARE MOST PRESENT AND MOST RELEVANT FOR THE RESPONDENT]

H. BACK

H1. Back:
Have you ever had a wound, burn, mass, deformity, or an operation on your back?
___ Yes
___ No
[If there were/are no problems with this anatomical section you can continue with the following. If the person had a problem with this anatomical section you question first on problem 1 all the questions on this page and go further if here are more problems]

H2.1. Back specifics:

Problem 1	Problem 2	Problem 3

Tell me what problem you have had.

Wound injury related
Wound not injury related

Institutional Review Board
American University of Beirut

10 MAY 2021

APPROVED

Burn
Mass or growth
Deformity congenital
Deformity acquired

[Wound: Open skin; sometimes leaking blood, pus or liquid
Deformity: An abnormal tissue arrangement, malformation
Congenital: The person is born with the problem.
Acquired: The person got the problem later in life]

H2.1.1 Type of injury / accident:
Did the problem started after an injury or accident? What kind of accident?

Problem 1	Problem 2	Problem 3

[Pick the one that best describes the injury / accident. Pedestrian and bicycle crash definition: there was no motorized vehicle involved. All can be intentional or unintentional.]

No, it was not due to an injury / accident
Car, truck, bus crash
Motorcycle crash
Pedestrian, bicycle crash
Gunshot
Stab / slash / cut / crush
Bite or animal attack
Fall
Open fire / explosion
Hot liquid / hot object

Problem 1	Problem 2	Problem 3

H3.1. Timing:
When did this problem start? In the last month
During the past 12 months but longer than a month ago
Longer than 12 months ago

H3.1.1 At this moment:

Problem 1	Problem 2	Problem 3

Do you have this problem now (or during the last week)? Yes
No

Problem 1	Problem 2	Problem 3

H4.1. Healthcare sought:
Did you go to a health facility or see a doctor/nurse for this problem? Yes
No

Problem 1	Problem 2	Problem 3

Institutional Review Board
American University of Beirut

10 MAY 2021

APPROVED

H4.1.1. Traditional Healer:
Did you go to a traditional healer, traditional doctor, or bone setter for this problem?

Yes
No

H5.1. Type of healthcare received: *[Only when 'Yes' to H4.1]*
What kind of treatment did you receive?

Problem 1	Problem 2	Problem 3

None / No surgical care
Major procedure = a procedure which requires regional/general anesthesia
Minor procedures = dressings, wound care, punctures, suturing and I&D

Institutional Review Board
American University of Beirut
10 MAY 2021
APPROVED

H6.1. Reason for not having surgical care: *[Only when 'none / no surgical care' to H5.1 or 'no' to H4.1]*
What was the main reason not to go to a health facility to see a doctor/nurse or not to have an operation or dressings?
[If the person went to a traditional healer ask why (s)he didn't go to a health care facility and mark that as the answer. If the person was referred but did not go to the referral hospital, mark the answer why (s)he did not go here.]

Problem 1	Problem 2	Problem 3

No money for health care
No (money for) transportation
No time
Fear / no trust
Not available (facility/personnel/equipment)
No need

H7.1. Disability:

Problem 1	Problem 2	Problem 3

Does this problem still impact your daily life?

The condition is not disabling
I feel ashamed
I'm not able to work like I used to
I need help with transportation
I need help with daily living

[Disability: a physical problem that impacts your life, or makes it difficult to carry out your daily activities.]

[ASK IF THE PERSON HAD ANOTHER PROBLEM REGARDING THIS ANATOMICAL LOCATION. IF SO, GO TO THE FOLLOWING ROW, PROBLEM 2 OR 3, TO REGISTER THIS PROBLEM. IF HERE ARE NO OTHER PROBLEMS REGARDING TO THIS LOCATION YOU CAN GO TO THE FOLLOWING ANATOMICAL LOCATION. IN CASE OF MORE THAN 3 PROBLEMS, MARK THE ONES WHICH ARE MOST PRESENT AND MOST RELEVANT FOR THE RESPONDENT]

I. ABDOMEN

I1. Abdomen:
Have you ever had a wound, burn, mass, deformity, inability to urinate, bleeding from your bottom, abdominal delivery, or an operation on your abdomen?
___ Yes
___ No
[If there were/are no problems with this anatomical section you can continue with the following. If the person had a problem with this anatomical section you question first on problem 1 all the questions on this page and go further if here are more problems]

Problem 1	Problem 2	Problem 3

Institutional Review Board
American University of Beirut

10 MAY 2021

APPROVED

I2.1. Abdomen specifics:
Tell me what problem you have had. Wound injury related

Wound not injury related
Burn
Mass or growth (solid)
Mass or growth (soft reducible)
Deformity congenital
Deformity acquired
Abdominal distention or pain
Inability to urinate
Bleeding (per rectum)
Bleeding(per penis)

I2.1.1 Type of injury / accident:
Did the problem started after an injury or accident? What kind of accident?

Problem 1	Problem 2	Problem 3

[Pick the one that best describes the injury / accident. Pedestrian and bicycle crash definition: there was no motorized vehicle involved. All can be intentional or unintentional.]

No, it was not due to an injury / accident
Car, truck, bus crash
Motorcycle crash
Pedestrian, bicycle crash
Gunshot
Stab / slash / cut / crush
Bite or animal attack
Fall
Open fire / explosion
Hot liquid / hot object

Problem 1	Problem 2	Problem 3

I3.1. Timing:
When did this problem start? In the last month
During the past 12 months but longer than a month ago
Longer than 12 months ago

Problem 1	Problem 2	Problem 3

I3.1.1 At this moment:
Do you have this problem now (or during the last week)? Yes
No

Problem 1	Problem 2	Problem 3

I4.1. Healthcare sought:
Did you go to a health facility or see a doctor/nurse for this problem? Yes
No

Institutional Review Board
American University of Beirut
10 MAY 2021
APPROVED

Problem 1	Problem 2	Problem 3

I4.1.1. Traditional Healer:

Did you go to a traditional healer, traditional doctor, or bone setter for this problem?

Yes

No

I5.1. Type of healthcare received: *[Only when 'Yes' to I4.1]*

What kind of treatment did you receive?

Problem 1	Problem 2	Problem 3

None / No surgical care

Major procedure = a procedure which requires regional/general anesthesia

Minor procedures = dressings, wound care, punctures, suturing and I&D

Cesarean section

[Cesarean section: Abdominal delivery. The baby is born with an abdominal operation.]

I6.1. Reason for not having surgical care: *[Only when 'none / no surgical care' to I5.1 or 'no' to I4.1]*

Problem 1	Problem 2	Problem 3

What was the main reason not to go to a health facility to see a doctor/nurse or not to have an operation or dressings?

[If the person went to a traditional healer ask why (s)he didn't go to a health care facility and mark that as the answer. If the person was referred but did not go to the referral hospital, mark the answer why (s)he did not go here.]

No money for health care

No (money for) transportation

No time

Fear / no trust

Not available (facility/personnel/equipment)

No need

I7.1. Disability:

Problem 1	Problem 2	Problem 3

Does this problem still impact your daily life?

The condition is not disabling

I feel ashamed

I'm not able to work like I used to

I need help with transportation

I need help with daily living

[Disability: a physical problem that impacts your life, or makes it difficult to carry out your daily activities.]

[ASK IF THE PERSON HAD ANOTHER PROBLEM REGARDING THIS ANATOMICAL LOCATION. IF SO, GO TO THE FOLLOWING ROW, PROBLEM 2 OR 3, TO REGISTER THIS PROBLEM. IF HERE ARE NO OTHER PROBLEMS REGARDING TO THIS LOCATION YOU CAN GO TO THE FOLLOWING ANATOMICAL LOCATION. IN CASE OF MORE THAN 3 PROBLEMS, MARK THE ONES WHICH ARE MOST PRESENT AND MOST RELEVANT FOR THE RESPONDENT]

Institutional Review Board
American University of Beirut

10 MAY 2021

APPROVED

J. GROIN / GENITALIA / BUTTOCKS (TAB: Survey Part III)

J1. Groin / genitalia / buttocks:
Have you ever had a wound, burn, mass, deformity, leaking of urine or feces, bleeding from your bottom, bleeding from your penis, or an operation on your groin, genitalia or buttocks?
___ Yes
___ No

Problem 1	Problem 2	Problem 3

J2.1. Groin / genitalia specifics:
Tell me what problem you have had.

Wound due to an injury
Wound not due to an injury
Burn
Mass or growth (solid) *[testicular cancer or hydrocele/cystocele]*
Mass (soft or reducible) *[inguinal hernia]*
Deformity congenital
Deformity acquired
Leaking of urine or feces (like fistula)
Bleeding (per rectum)
Bleeding (from the penis)

[Deformity: An abnormal tissue arrangement, malformation
Congenital: The person is born with the problem. Think about: born without anus, hypospadias etc.
Acquired: The person got the problem later in life]

J2.1.1 Type of injury / accident:
Did the problem started after an injury or accident? What kind of accident?

Problem 1	Problem 2	Problem 3

[Pick the one that best describes the injury / accident. Pedestrian and bicycle crash definition: there was no motorized vehicle involved. All can be intentional or unintentional.]

No, it was not due to an injury / accident
Car, truck, bus crash
Motorcycle crash
Pedestrian, bicycle crash
Gunshot
Stab / slash / cut / crush
Bite or animal attack
Fall
Open fire / explosion
Hot liquid / hot object

Problem 1	Problem 2	Problem 3

Institutional Review Board
American University of Beirut

10 MAY 2021

APPROVED

J3.1. Timing:

When did this problem start?

In the last month During the past 12 months but longer than a month ago

Longer than 12 months ago

J3.1.1 At his moment:

Problem 1	Problem 2	Problem 3

Do you have this problem now (or during the last week)? Yes

No

Problem 1	Problem 2	Problem 3

J4.1. Healthcare sought:

Did you go to a health facility or see a doctor/nurse for this problem? Yes

No

Problem 1	Problem 2	Problem 3

J4.1.1. Traditional Healer:

Did you go to a traditional healer, traditional doctor, or bone setter for this problem? Yes

No

J5.1. Type of healthcare received: *[Only when 'Yes' to J4.1]*

Problem 1	Problem 2	Problem 3

What kind of treatment did you receive?

None / No surgical care

Major procedure = a procedure which requires regional/general anesthesia

Minor procedures = dressings, wound care, punctures, suturing and I&D

Institutional Review Board
American University of Beirut

10 MAY 2021

APPROVED

J6.1. Reason for not having surgical care: *[Only when 'none / no surgical care' to J5.1 or 'no' to J4.1]*
What was the main reason not to go to a health facility to see a doctor/nurse or not to have an operation or dressings?
[If the person went to a traditional healer ask why (s)he didn't go to a health care facility and mark that as the answer. If the person was referred but did not go to the referral hospital, mark the answer why (s)he did not go here.]

Problem 1	Problem 2	Problem 3

No money for health care
No (money for) transportation
No time
Fear / no trust
Not available (facility/personnel/equipment)
No need

J7.1. Disability:

Problem 1	Problem 2	Problem 3

Does this problem still impact your daily life?

The condition is not disabling
I feel ashamed
I'm not able to work like I used to
I need help with transportation
I need help with daily living

[Disability: a physical problem that impacts your life, or makes it difficult to carry out your daily activities.]

[ASK IF THE PERSON HAD ANOTHER PROBLEM REGARDING THIS ANATOMICAL LOCATION. IF SO, GO TO THE FOLLOWING ROW, PROBLEM 2 OR 3, TO REGISTER THIS PROBLEM. IF HERE ARE NO OTHER PROBLEMS REGARDING TO THIS LOCATION YOU CAN GO TO THE FOLLOWING ANATOMICAL LOCATION. IN CASE OF MORE THAN 3 PROBLEMS, MARK THE ONES WHICH ARE MOST PRESENT AND MOST RELEVANT FOR THE RESPONDENT]

K. EXTREMITIES

K1. Extremities:
Have you ever had an injury, burn, wound, mass, deformity, broken bone, or an operation on your hands, feet, arms, or legs?
___ Yes
___ No
[If there were/are no problems with this anatomical section you can continue with the following. If the person had a problem with this anatomical section you question first on problem 1 all the questions on this page and go further if here are more problems]

Problem 1	Problem 2	Problem 3

K2.1. Extremity location:
On what part of your body did the problem occur?

Finger(s)
Thumb / Hand
Lower arm
Upper arm
Foot
Lower leg
Upper leg

Institutional Review Board
American University of Beirut
10 MAY 2021
APPROVED

[If the problem is right and left: make two separate tabs to document the problem.]
[If the problem is based on a joint you need to choose the most proximate location to the abdomen.
For example: an elbow fracture is documented as the upper arm, a knee skin contracture is documented as the upper leg.]

K3.1. Extremity specifics:

Problem 1	Problem 2	Problem 3

Tell me what problem you have had.

Wound injury related
Wound not injury related
(Recurrent) drainage / discharge
Burn
Mass growth
Deformity congenital
Deformity acquired

[Recurrent) drainage / discharge from small sore or opening in the skin: a high suspicion for osteomyelitis, infected bone
Deformity: An abnormal tissue arrangement, malformation
Congenital: The person is born with the problem. Think about: clubfeet
Acquired: The person got the problem later in life. Think about: broken bones and scar contractures]

Institutional Review Board
American University of Beirut

10 MAY 2021

APPROVED

K3.1.1 Type of injury / accident:
Did the problem started after an injury or accident? What kind of accident?

Problem 1	Problem 2	Problem 3

[Pick the one that best describes the injury / accident. Pedestrian and bicycle crash definition: there was no motorized vehicle involved. All can be intentional or unintentional.]

No, it was not due to an injury / accident
Car, truck, bus crash
Motorcycle crash
Pedestrian, bicycle crash
Gunshot
Stab / slash / cut / crush
Bite or animal attack
Fall
Open fire / explosion
Hot liquid / hot object

K4.1. Timing:

Problem 1	Problem 2	Problem 3

When did this problem start?

In the last month
During the past 12 months but longer than a month ago
Longer than 12 months ago

Problem 1	Problem 2	Problem 3

K4.1.1 At this moment:
Do you have this problem now (or during the last week)? Yes
No

Problem 1	Problem 2	Problem 3

K5.1. Fracture:
Did you break a bone or dislocate a joint? Yes
No

Problem 1	Problem 2	Problem 3

K6.1. Healthcare sought:
Did you go to a health facility or see a doctor/nurse for this problem? Yes
No

Problem 1	Problem 2	Problem 3

K6.1.1. Traditional Healer:
Did you go to a traditional healer, traditional doctor, Yes

Institutional Review Board
American University of Beirut
10 MAY 2021
APPROVED

or bone setter for this problem? No

K7.1. Type of healthcare received: *[Only when 'Yes' to K6.1]*

What kind of treatment did you receive?

Problem 1	Problem 2	Problem 3

None / No surgical care
Major procedure = a procedure which requires regional/general anesthesia
Minor procedures = dressings, wound care, punctures, suturing and I&D Manipulation / casting / sling
Traction

[Internal / external fixation: the bone is fixed with an operation. Sometimes the metal needs to be removed afterwards.
Manipulation / casting / sling: the broken bone was immobilized for some time.
Traction: the patient needed to stay in a hospital bed for a long time, the broken limb was fixed to the bed or a heavy object.]

K8.1. Reason for not having surgical care: *[Only when 'none / no surgical care' to K7.1 or 'no' to K6.1]*

What was the main reason not to go to a health facility to see a doctor/nurse or not to have an operation or dressings?

Problem 1	Problem 2	Problem 3

[If the person went to a traditional healer ask why (s)he didn't go to a health care facility and mark that as the answer.
If the person was referred but did not go to the referral hospital, mark the answer why (s)he did not go here.]

No money for health care
No (money for) transportation
No time
Fear / no trust
Not available (facility/personnel/equipment)
No need

K9.1. Disability:

Problem 1	Problem 2	Problem 3

Does this problem still impact your daily life?

The condition is not disabling
I feel ashamed
I'm not able to work like I used to
I need help with transportation
I need help with daily living

[Disability: a physical problem that impacts your life, or makes it difficult to carry out your daily activities.]

[ASK IF THE PERSON HAD ANOTHER PROBLEM REGARDING THIS ANATOMICAL LOCATION. IF SO, GO TO THE FOLLOWING COLLUMN, PROBLEM 2 AND/OR 3, TO REGISTER THIS PROBLEM. IF HERE ARE NO OTHER PROBLEMS REGARDING TO THIS LOCATION YOU CAN GO TO THE FOLLOWING ANATOMICAL LOCATION. IN CASE OF MORE THAN 3 PROBLEMS, MARK THE ONES WHICH ARE MOST PRESENT AND MOST RELEVANT FOR THE RESPONDENT]

Institutional Review Board
American University of Beirut

10 MAY 2021

APPROVED

L. WOMEN'S HEALTH

Reproductive age demographic screening:

L1. Are you currently at a reproductive age?
1. No
2. Yes
If yes, age:
IF ABOVE REPRODUCTIVE AGE, ASK UNTIL ITEM L14 included, ask L18, and skip to L34. If 'MALE' OR 'GIRL UNDER THE AGE OF 12 YEARS' YOU CAN SKIP ALL THE FOLLOWING QUESTIONS AND GO TO THE END OF THIS FORM TO FINISH THE SURVEY WITH A LAST CHECK OF THE SURVEY AND YOUR SIGNATURE]

L2. Educational level:
1. No education
2. Some school education
3. Baccalaureate degree
4. Higher education

L3. Smoking status:
1. Smoker
2. Never
3. Previous smoker

L4. Primary source (s) of income:
1. No income
2. Spouse
3. Family other than husband
4. Self
5. Charity/assistance

L5. Registered refugee?
1. No
2. Yes

L6. Marital Status:
1. Single
2. Married
3. Divorced
4. Widowed

L7. Age at first marriage: ____

L8. Consanguineous marriage?
1. No
2. Yes

Reproductive health screening:

L9. During your stay in the camp, have you recurrently had infections in the urinary tract?
1. No
2. Yes, self-assessment
3. Yes, evaluated by a professional

If answer if yes, continue with question L10, otherwise skip to L11

L10. If yes, did you receive treatment?
1. No
2. Yes

L11. During your stay in the camp, have you had any gynecologic problems?
1. Irregular bleeding
2. Genital tract infection
3. Severe pain in pelvic/genital area
4. Severe pain during menses (dysmenorrhea)
5. None

If answer includes "Genital tract infection", continue with question L12, otherwise skip to L13

L12. If genital tract infection, where you evaluated by a professional?
1. No
2. Yes

Institutional Review Board
American University of Beirut

10 MAY 2021

APPROVED

L13. Are sanitary pads available to you?
1. No, no access
2. No, can't afford
3. Yes
4. I don't need them

If answer is yes, ask L14, otherwise skip to L15

L14. How many pads/towels do you use per day, on your heaviest bleeding days? _____

_____
L15. Do you realize any bleeding between two periods?
1. No
2. Yes

Gravida
L16. Have you had any pregnancy during camp stay?
1. No
2. Yes, ______

L20. **Have you had any miscarriages during camp stay?**
1. No
2. Yes, ______

If no, skip to L19, and then L34.

Pregnancy:
L18. Are you currently pregnant?
___ Yes
___ No
___ I don't know

L19. Age at first pregnancy: ____

L20. Smoking during pregnancy:
1. No
2. Yes

Obstetric complications and obstetric care capacity:

These questions are in regard to the latest pregnancy in the camp, except L23:

L21. Have you had any antenatal Care visit with a skilled professional during your pregnancy?
1. No
2. Yes, ______

L22. Did you face severe infections during pregnancy and childbirth requiring medical attention?
1. No
2. Yes
If "yes", ask L22' otherwise skip to L23.

L22'. If yes, did you seek medical care?
1. No
2. Yes
If "no", ask L22'' otherwise skip to L23.

L22''. If no, reason:
1. No transportation
2. No money for surgery
3. No time (died before they reached)
4. Fear/No trust
5. No facility available

L23. On any of your pregnancies, have your had hypertension during pregnancy which wasn't present prior, diagnosed by a healthcare professional?
1. No
2. Yes
If "yes", ask L23' otherwise skip to L24.

Institutional Review Board
American University of Beirut

10 MAY 2021

APPROVED

L23'. If yes, did you seek medical care?
1. No
2. Yes

Delivery and obstetric care capacity:

L24. How was the delivery?
1. NVD at home
3. NVD in medical facility (clinic or hospital)
4. C-section in a clinic within the camp
5. C-section in a medical facility (clinic or hospital)

L25. If at home, was there any skilled birth attendance (midwife/nurse, doctor)
1. No
2. Yes

L26. Did you have excess bleeding during delivery?
1. No
2. Yes

L27. Did you receive any postpartum care?
1. No
2. Yes, midwife at home
3. Yes, at clinic

Family Planning:

L28. Did you breastfeed your babies?
1. No
2. Yes, _____ months

L29. Would you like to prevent pregnancy?
1. No
2. Yes

L30. Access to contraception?
1. No
2. Yes

L31**. If yes, method used?**
1. IUD
2. Condom
3. OCP
4. Injection
5. Withdrawal
6. Menstrual calendar

L32. Have you ever discussed family planning with a professional?
1. No
2. Yes

L33. If yes, who was this professional?
1. Doctor
2. Nurse
3. Midwife
4. Relative

Perceptions of healthcare accessibility:

L34. What is your perception of overall reproductive health accessibility?
1. Easily accessible
2. Inaccessible
3. Difficult to access
4. Not sure

L35. What is your perception of overall medical services accessibility?
1. Easily accessible
2. Inaccessible
3. Difficult to access
4. Not sure

Institutional Review Board
American University of Beirut

10 MAY 2021

APPROVED

Thank you very much for answering these questions. Let me check everything.

[Go over all the tabs to ensure that you have everything. Ask the questions again which you accidentally skipped.

I checked the full Survey, there is no data missing:
______________ *[date]* _______________ *[name]* _______________ *[signature of interviewer]*

I see that I have everything that I need; your survey is complete. Thanks again on behalf of the research team. Do you have questions for me? You are always welcome to contact us. Have a nice day.

[Give or refer to the letter with the (contact) information about the survey.] [TIME: ____ hour ____ min]

If you wish to make a comment for this interview you can do that below:
____
____
____
____

Institutional Review Board
American University of Beirut

10 MAY 2021

APPROVED

E. GENERAL INFORMATION (Survey 2) SECOND RESPONDENT [TIME: ____ hour ____ min]

Ex1. Household list (ID) number: _____

[from the table with the questions B1 and B2]
[Check this box if this is a replaced household member] ☐

[If a surrogate is answering for a child, ask all the questions about the child.]

Ex2. Sex:
___ Male
___ Female

Ex3. Age:
____

[Age for babies:

<3 months	*= 0*
3- <6 months	*= 0.25*
6- <9 months	*= 0.5*
9- <12 months	*= 0.75*
12-24 months	*= 1 etc.]*

'Good morning/evening. My name is _______________. I am helping with a project from the American University in Beirut. We are trying to find out if there are enough doctors in this area, specifically if there are enough surgeons. A surgeon is a medical doctor who cures patients by taking care of wounds and broken bones or cutting out masses. Sometimes surgeons must put you to sleep to do these things, and other times they must only numb the hurt body part. The purpose of this study is to find out more what the problems are and the reasons it may be hard to receive care.

To find out if there are enough doctors taking care of these problems in this area, we'd like to ask you and some other members of your household some questions. We will ask questions about health, such as whether members of your household have ever had wounds, broken bones, or masses. By asking these questions, we hope that we can help make access to surgery more easy in this area. We won't be offering medical care right now, but we hope that the information you provide will help create improved services in the future.

This survey will take about 30 minutes to 1 hour. All of your responses will be confidential. You have the right to not participate in the survey, or to stop during the interview. First I will ask you some questions about the people who live in this house. After that, I will randomly choose two people from your household and ask them more detailed questions about their health. This information is confidential, and nobody will find out what answers you gave other than me and my research team and we will not collect your name or address. We have an information sheet for you, and we will obtain your consent to participate and separate from the household members who will participate. Do you have any questions at this moment?

Ex4. Informed consent: Would you like to participate in this survey? OR
Is it okay for your son/daughter to participate in this survey?
___ Yes
___ No, what is the reason? (no time / no willingness / no reason / no seen benefit / other: explain….)

[For minors (individuals under age 18), this consent E4 is obtained from a guardian/parent. Without informed consent you cannot proceed. Make sure the person understands the purpose of this survey. If they don't want to participate, ask why and mark this.]

Ex5. Minor assent: *[under 18 years of age only]* Would you like to participate in this survey?
___ Yes
___ No, what is the reason? (no time / no willingness / no reason / no seen benefit / other: explain….)
___ Surrogate consent only

[Surrogate consent only: this can be used in case of a child under 12 years of age, who is not around for the interview. The guardian/parent can answer all the questions for the child.
When the child is around, the permission to participate (assent) is asked and only with the assent of the child the interview is held together with the guardian/parent. For children over the age of 12 the guardian/parent can be around for the interview depending on the wish of the child.]

The following questions are general questions, later on I will ask more about your health.

Ex6. Education:
What is the highest educational level that you have achieved or are currently following?
___ None (includes nursery)
___ Primary school
___ Secondary school (junior / senior)
___ Tertiary (diploma, colleges, bachelors)
___ Graduate degree (Master degree, PhD)

Ex6.1 Literacy:
Are you able to read and write in any language?
[For adults and children who are currently learning how to read and write answer: 'No']

Institutional Review Board
American University of Beirut
10 MAY 2021
APPROVED

___ Yes
___ No

Institutional Review Board
American University of Beirut

10 MAY 2021

APPROVED

Ex7. Occupation:
What is your primary occupation?

___ Unemployed	*[Currently looking for jobs, retiree's, students]*
___ Home maker	*[Housewives]*
___ Domestic helpers	*[Cleaners, housekeepers, watch guards]*
___ Farmer	*[Herders, agriculture, pastoralist]*
___ Self-employed / small-business	*[Small business owners like: shops, kiosks, food traders]*
___ Government employee	*[Police officer, accountant, teachers, health care workers]*
___ Non-government employee	*[Cooperation managers, NGO-staff]*

E8. Origin?
What is your ethnic background?
_____ Syrian
_____ Palestinian
_____ Iraqi
_____ Kurdish
_____ Yemeni
_____ Lebanese
_____ Egyptian
_____ Other: ___________

E 8.1 Syrian Origin?
If answered [Yes] to Syrian, where in Syrian are you from?
_____ Damascus
_____ Aleppo
_____ Idlib
_____ Latakia
_____ Tartus
_____ Eastern Ghouta
_____ Western Ghouta

Ex9. Length of stay in house:
How many years have you lived in this household?
_____ *[years]*

Ex10. Health status: [TIME: ____ hour ____ min]
Are you generally healthy?
___ Yes
___ No
[if 'Yes' to E10. The following questions E11, E12 and E13, can be skipped]

Ex11. Time ill:
In total how many weeks have you been ill during the past year?
_____ *[weeks]*

Ex12. Number of health facility visits:
How many times have you visited a clinic or hospital, or nurse / medical doctor in the last year?
_____

Ex13. Recovery from illness:
Have you recovered fully from the illness you had?
___ Yes
___ No

EXPLAIN:

Surgery, also known as an operation, can be done for a swelling, mass, abdominal pain, and many other things. Patients often have a bandage after having surgery or may need to stay in the hospital for some time. Sometimes, children are born with problems that can be fixed with an operation. Examples of these problems are open lips, missing anus, or strange feet.

Some people who break a bone or have a wound, may not have an operation but still need to be seen by a doctor or stay in the surgical ward of a hospital. Since this does not include an operation, but includes surgical consultation, we call it surgical care.

Now I'm going to ask you about all the surgical problems you've had in your lifetime. We'll start with your head and move all the way down to your toes.

Institutional Review Board
American University of Beirut
10 MAY 2021
APPROVED

F. FACE / HEAD / NECK

Fx1. Face / head / neck:
Have you ever had a wound, burn, mass / goiter, deformity, problem with eating/drinking, a problem with your eyes or ears or an operation on your face, head, or neck?
___ Yes
___ No

[IF THERE WERE/ARE NO PROBLEMS WITH THIS ANATOMICAL SECTION YOU CAN CONTINUE WITH SECTION G. IF THE PERSON HAD A PROBLEM WITH THIS ANATOMICAL SECTION YOU QUESTION FIRST ON PROBLEM 1 ALL THE QUESTIONS ON THIS PAGE AND GO FURTHER IF HERE ARE MORE PROBLEMS]

Fx1.1. Face / head / neck location:

Problem 1	Problem 2	Problem 3

On what part of your head / neck / face did the problem occur?

Eye
Ear / nose / throat
Dental / lips / mouth
Neck
Head

Fx2.1. Face / head / neck specifics:

Problem 1	Problem 2	Problem 3

Tell me what problem you have had.

Wound injury related
Wound not injury related
Burn
Mass or growth / goiter
Deformity congenital
Deformity acquired

[Wound: Open skin; sometimes leaking blood, pus or liquid
Deformity: An abnormal tissue arrangement or malformation
Congenital: The person is born with the problem. Think about: cleft lips, hydrocephalus etc.
Acquired: The person got the problem later in life. Think about: scars and broken bones]

Fx2.1.1 Type of injury / accident:
Did the problem started after an injury or accident? What kind of accident?

Problem 1	Problem 2	Problem 3

[Pick the one that best describes the injury / accident. Pedestrian and bicycle crash definition: there was no motorized vehicle involved. All can be intentional or unintentional.]

No, it was not due to an injury / accident
Car, truck, bus crash
Motorcycle crash
Pedestrian, bicycle crash
Gunshot
Stab / slash / cut / crush

Institutional Review Board
American University of Beirut

10 MAY 2021

APPROVED

Bite or animal attack
Fall
Open fire / explosion
Hot liquid / hot object

Problem 1	Problem 2	Problem 3

Fx3.1. Timing:
When did this problem start?

In the last month
During the past 12 months but longer than a month ago
Longer than 12 months ago

Problem 1	Problem 2	Problem 3

Fx3.1.1 At this moment:
Do you have this problem now (or during the last week)?

Yes
No

Problem 1	Problem 2	Problem 3

Fx4.1. Healthcare sought:

Did you go to a health facility or see a doctor/nurse for this problem? Yes No

Problem 1	Problem 2	Problem 3

Fx4.1.1. Traditional Healer:
Did you go to a traditional healer, traditional doctor, or bone setter for this problem?

Yes
No

Institutional Review Board
American University of Beirut

10 MAY 2021

APPROVED

Fx5.1. Type of healthcare received: *[Only when 'yes' to F4.1]*
What kind of treatment did you receive?

Problem 1	Problem 2	Problem 3

None / No surgical care

Major procedure = a procedure which requires regional/general anesthesia

Minor procedures = dressings, wound care, punctures, suturing and I&D

Fx6.1. Reason for not having surgical care: *[Only when 'none / no surgical care' to F5.1 or 'no' to F4.1]*
What was the main reason not to go to a health facility to see a doctor/nurse or not to have an operation or dressings?
[If the person went to a traditional healer ask why (s)he didn't go to a health care facility and mark that as the answer. If the person was referred but did not go to the referral hospital, mark the answer why (s)he did not go here.]

Problem 1	Problem 2	Problem 3

No money for health care
No (money for) transportation
No time
Fear / no trust
Not available (facility/personnel/equipment)
No need

Fx7.1. Disability:

Problem 1	Problem 2	Problem 3

Does this problem still impact your daily life?

The condition is not disabling
I feel ashamed
I'm not able to work like I used to
I need help with transportation
I need help with daily living

[Disability: a physical problem that impacts your life, or makes it difficult to carry out your daily activities.]

[ASK IF THE PERSON HAD ANOTHER PROBLEM REGARDING THIS ANATOMICAL LOCATION. IF SO, GO TO THE FOLLOWING ROW, PROBLEM 2 OR 3, TO REGISTER THIS PROBLEM. IF HERE ARE NO OTHER PROBLEMS REGARDING TO THIS LOCATION YOU CAN GO TO THE FOLLOWING ANATOMICAL LOCATION. IN CASE OF MORE THAN 3 PROBLEMS, MARK THE ONES WHICH ARE MOST PRESENT AND MOST RELEVANT FOR THE RESPONDENT]

G. CHEST / BREAST (TAB: Survey Part II)

Gx1. Chest / breast:
Have you ever had a wound, burn, breast mass, deformity, or an operation on your chest (including heart or lungs) or breast?
___ Yes
___ No

[IF THERE WERE/ARE NO PROBLEMS WITH THIS ANATOMICAL SECTION YOU CAN CONTINUE WITH THE FOLLOWING. IF THE PERSON HAD A PROBLEM WITH THIS ANATOMICAL SECTION YOU QUESTION FIRST ON PROBLEM 1 ALL THE QUESTIONS ON THIS PAGE AND GO FURTHER IF HERE ARE MORE PROBLEMS]

Gx2.1. Chest / breast specifics:

Problem 1	Problem 2	Problem 3

Institutional Review Board
American University of Beirut

10 MAY 2021

APPROVED

Tell me what problem you have had.

Wound injury related
Wound not injury related
Burn
Breast mass / breast cancer
Deformity congenital
Deformity acquired

[Wound: *Open skin; sometimes leaking blood, pus or liquid*
Deformity: *An abnormal tissue arrangement, malformation*
Congenital: *The person is born with the problem. Example: heart malformation*
Acquired: *The person got the problem later in life]*

Institutional Review Board
American University of Beirut

10 MAY 2021

APPROVED

Gx2.1.1 Type of injury / accident:
Did the problem started after an injury or accident? What kind of accident?

Problem 1	Problem 2	Problem 3

[Pick the one that best describes the injury / accident. Pedestrian and bicycle crash definition: there was no motorized vehicle involved. All can be intentional or unintentional.]

No, it was not due to an injury / accident
Car, truck, bus crash
Motorcycle crash
Pedestrian, bicycle crash
Gunshot
Stab / slash / cut / crush
Bite or animal attack
Fall
Open fire / explosion
Hot liquid / hot object

Problem 1	Problem 2	Problem 3

Gx3.1. Timing:
When did this problem start?

In the last month
During the past 12 months but longer than a month ago
Longer than 12 months ago

Problem 1	Problem 2	Problem 3

Gx3.1.1 At this moment:
Do you have this problem now (or during the last week)? Yes
No

Problem 1	Problem 2	Problem 3

Gx4.1. Healthcare sought:
Did you go to a health facility or see a doctor/nurse for this problem? Yes
No

Problem 1	Problem 2	Problem 3

Gx4.1.1. Traditional Healer:
Did you go to a traditional healer, traditional doctor, Yes
or bone setter for this problem? No

Problem 1	Problem 2	Problem 3

Institutional Review Board
American University of Beirut

10 MAY 2021

APPROVED

Gx5.1. Type of healthcare received: *[Only when 'Yes' to G4.1]*
What kind of treatment did you receive?

None / No surgical care
Major procedure = a procedure which requires regional/general anesthesia
Minor procedures = dressings, wound care, punctures, suturing and I&D

Gx6.1. Reason for not having surgical care: *[Only when 'none / no surgical care' to G5.1 or 'no' to G4.1]*
What was the main reason not to go to a health facility to see a doctor/nurse or not to have an operation or dressings?
[If the person went to a traditional healer ask why (s)he didn't go to a health care facility and mark that as the answer. If the person was referred but did not go to the referral hospital, mark the answer why (s)he did not go here.]

Problem 1	Problem 2	Problem 3

No money for health care
No (money for) transportation
No time
Fear / no trust
Not available (facility/personnel/equipment)
No need

Gx7.1. Disability:

Problem 1	Problem 2	Problem 3

Does this problem still impact your daily life?

The condition is not disabling
I feel ashamed
I'm not able to work like I used to
I need help with transportation
I need help with daily living

[Disability: a physical problem that impacts your life, or makes it difficult to carry out your daily activities.]
[ASK IF THE PERSON HAD ANOTHER PROBLEM REGARDING THIS ANATOMICAL LOCATION. IF SO, GO TO THE FOLLOWING ROW, PROBLEM 2 OR 3, TO REGISTER THIS PROBLEM. IF HERE ARE NO OTHER PROBLEMS REGARDING TO THIS LOCATION YOU CAN GO TO THE FOLLOWING ANATOMICAL LOCATION. IN CASE OF MORE THAN 3 PROBLEMS, MARK THE ONES WHICH ARE MOST PRESENT AND MOST RELEVANT FOR THE RESPONDENT]

H. BACK

Hx1. Back:
Have you ever had a wound, burn, mass, deformity, or an operation on your back?
___ Yes
___ No
[If there were/are no problems with this anatomical section you can continue with the following. If the person had a problem with this anatomical section you question first on problem 1 all the questions on this page and go further if here are more problems]

Hx2.1. Back specifics:

Problem 1	Problem 2	Problem 3

Tell me what problem you have had.

Wound injury related
Wound not injury related

Institutional Review Board
American University of Beirut

10 MAY 2021

APPROVED

Burn
Mass or growth
Deformity congenital
Deformity acquired

[Wound: Open skin; sometimes leaking blood, pus or liquid
Deformity: An abnormal tissue arrangement, malformation
Congenital: The person is born with the problem.
Acquired: The person got the problem later in life]

Hx2.1.1 Type of injury / accident:
Did the problem started after an injury or accident? What kind of accident?

Problem 1	Problem 2	Problem 3

[Pick the one that best describes the injury / accident. Pedestrian and bicycle crash definition: there was no motorized vehicle involved. All can be intentional or unintentional.]

No, it was not due to an injury / accident
Car, truck, bus crash
Motorcycle crash
Pedestrian, bicycle crash
Gunshot
Stab / slash / cut / crush
Bite or animal attack
Fall
Open fire / explosion
Hot liquid / hot object

Problem 1	Problem 2	Problem 3

Hx3.1. Timing:
When did this problem start? In the last month
During the past 12 months but longer than a month ago
Longer than 12 months ago

Hx3.1.1 At this moment:

Problem 1	Problem 2	Problem 3

Do you have this problem now (or during the last week)? Yes
No

Problem 1	Problem 2	Problem 3

Hx4.1. Healthcare sought:
Did you go to a health facility or see a doctor/nurse for this problem? Yes
No

Problem 1	Problem 2	Problem 3

Institutional Review Board
American University of Beirut

10 MAY 2021

APPROVED

Hx4.1.1. Traditional Healer:
Did you go to a traditional healer, traditional doctor, or bone setter for this problem? Yes

No

Hx5.1. Type of healthcare received: *[Only when 'Yes' to H4.1]*
What kind of treatment did you receive?

Problem 1	Problem 2	Problem 3

None / No surgical care
Major procedure = a procedure which requires regional/general anesthesia
Minor procedures = dressings, wound care, punctures, suturing and I&D

Institutional Review Board
American University of Beirut

10 MAY 2021

APPROVED

Hx6.1. Reason for not having surgical care: *[Only when 'none / no surgical care' to H5.1 or 'no' to H4.1]*
What was the main reason not to go to a health facility to see a doctor/nurse or not to have an operation or dressings?
[If the person went to a traditional healer ask why (s)he didn't go to a health care facility and mark that as the answer.
If the person was referred but did not go to the referral hospital, mark the answer why (s)he did not go here.]

Problem 1	Problem 2	Problem 3

No money for health care
No (money for) transportation
No time
Fear / no trust
Not available (facility/personnel/equipment)
No need

Hx7.1. Disability:

Problem 1	Problem 2	Problem 3

Does this problem still impact your daily life?

The condition is not disabling
I feel ashamed
I'm not able to work like I used to
I need help with transportation
I need help with daily living

[Disability: a physical problem that impacts your life, or makes it difficult to carry out your daily activities.]

[ASK IF THE PERSON HAD ANOTHER PROBLEM REGARDING THIS ANATOMICAL LOCATION. IF SO, GO TO THE FOLLOWING ROW, PROBLEM 2 OR 3, TO REGISTER THIS PROBLEM. IF HERE ARE NO OTHER PROBLEMS REGARDING TO THIS LOCATION YOU CAN GO TO THE FOLLOWING ANATOMICAL LOCATION. IN CASE OF MORE THAN 3 PROBLEMS, MARK THE ONES WHICH ARE MOST PRESENT AND MOST RELEVANT FOR THE RESPONDENT]

I. ABDOMEN

Ix1. Abdomen:
Have you ever had a wound, burn, mass, deformity, inability to urinate, bleeding from your bottom, abdominal delivery, or an operation on your abdomen?
___ Yes
___ No
[If there were/are no problems with this anatomical section you can continue with the following. If the person had a problem with this anatomical section you question first on problem 1 all the questions on this page and go further if here are more problems]

Problem 1	Problem 2	Problem 3

Institutional Review Board
American University of Beirut

10 MAY 2021

APPROVED

Ix2.1. Abdomen specifics:
Tell me what problem you have had. Wound injury related
Wound not injury related
Burn
Mass or growth (solid)
Mass or growth (soft reducible)
Deformity congenital
Deformity acquired
Abdominal distention or pain
Inability to urinate
Bleeding (per rectum)
Obstructed delivery

Ix2.1.1 Type of injury / accident:
Did the problem started after an injury or accident? What kind of accident?

Problem 1	Problem 2	Problem 3

[Pick the one that best describes the injury / accident. Pedestrian and bicycle crash definition: there was no motorized vehicle involved. All can be intentional or unintentional.]

No, it was not due to an injury / accident
Car, truck, bus crash
Motorcycle crash
Pedestrian, bicycle crash
Gunshot
Stab / slash / cut / crush
Bite or animal attack
Fall
Open fire / explosion
Hot liquid / hot object

Problem 1	Problem 2	Problem 3

Ix3.1. Timing:
When did this problem start? In the last month
During the past 12 months but longer than a month ago
Longer than 12 months ago

Problem 1	Problem 2	Problem 3

Ix3.1.1 At this moment:
Do you have this problem now (or during the last week)? Yes
No

Problem 1	Problem 2	Problem 3

Ix4.1. Healthcare sought:
Did you go to a health facility or see a doctor/nurse for this problem? Yes
No

Institutional Review Board
American University of Beirut

10 MAY 2021

APPROVED

Problem 1	Problem 2	Problem 3

Ix4.1.1. Traditional Healer:
Did you go to a traditional healer, traditional doctor, or bone setter for this problem?

Yes

No

Ix5.1. Type of healthcare received: *[Only when 'Yes' to I4.1]*
What kind of treatment did you receive?

Problem 1	Problem 2	Problem 3

None / No surgical care
Major procedure = a procedure which requires regional/general anesthesia
Minor procedures = dressings, wound care, punctures, suturing and I&D
Cesarean section

[Cesarean section: Abdominal delivery. The baby is born with an abdominal operation.]

Ix6.1. Reason for not having surgical care: *[Only when 'none / no surgical care' to I5.1 or 'no' to I4.1]*

Problem 1	Problem 2	Problem 3

What was the main reason not to go to a health facility to see a doctor/nurse or not to have an operation or dressings?
[If the person went to a traditional healer ask why (s)he didn't go to a health care facility and mark that as the answer. If the person was referred but did not go to the referral hospital, mark the answer why (s)he did not go here.]

No money for health care
No (money for) transportation
No time
Fear / no trust
Not available (facility/personnel/equipment)
No need

Ix7.1. Disability:

Problem 1	Problem 2	Problem 3

Does this problem still impact your daily life?

The condition is not disabling
I feel ashamed
I'm not able to work like I used to
I need help with transportation
I need help with daily living

[Disability: a physical problem that impacts your life, or makes it difficult to carry out your daily activities.]

[ASK IF THE PERSON HAD ANOTHER PROBLEM REGARDING THIS ANATOMICAL LOCATION. IF SO, GO TO THE FOLLOWING ROW, PROBLEM 2 OR 3, TO REGISTER THIS PROBLEM. IF HERE ARE NO OTHER PROBLEMS REGARDING TO THIS LOCATION YOU CAN GO TO THE FOLLOWING ANATOMICAL LOCATION. IN CASE OF MORE THAN 3 PROBLEMS, MARK THE ONES WHICH ARE MOST PRESENT AND MOST RELEVANT FOR THE RESPONDENT]

Institutional Review Board
American University of Beirut

10 MAY 2021

APPROVED

J. GROIN / GENITALIA / BUTTOCKS (TAB: Survey Part III)

Jx1. Groin / genitalia / buttocks:
Have you ever had a wound, burn, mass, deformity, leaking of urine or feces, bleeding from your bottom, bleeding from your penis, or an operation on your groin, genitalia or buttocks?
___ Yes
___ No

Problem 1	Problem 2	Problem 3

Jx2.1. Groin / genitalia specifics:
Tell me what problem you have had.

Wound due to an injury
Wound not due to an injury
Burn
Mass or growth (solid) *[testicular cancer or hydrocele/cystocele]*
Mass (soft or reducible) *[inguinal hernia]*
Deformity congenital
Deformity acquired
Leaking of urine or feces (like fistula)
Bleeding (per rectum)
Bleeding (from the penis)

[Deformity: An abnormal tissue arrangement, malformation
Congenital: The person is born with the problem. Think about: born without anus, hypospadias etc.
Acquired: The person got the problem later in life]

Jx2.1.1 Type of injury / accident:
Did the problem started after an injury or accident? What kind of accident?

Problem 1	Problem 2	Problem 3

[Pick the one that best describes the injury / accident. Pedestrian and bicycle crash definition: there was no motorized vehicle involved. All can be intentional or unintentional.]

No, it was not due to an injury / accident
Car, truck, bus crash
Motorcycle crash
Pedestrian, bicycle crash
Gunshot
Stab / slash / cut / crush
Bite or animal attack
Fall
Open fire / explosion
Hot liquid / hot object

Problem 1	Problem 2	Problem 3

Institutional Review Board
American University of Beirut

10 MAY 2021

APPROVED

Jx3.1. Timing:

When did this problem start?

In the last month During the past 12 months but longer than a month ago

Longer than 12 months ago

Jx3.1.1 At his moment:

Problem 1	Problem 2	Problem 3

Do you have this problem now (or during the last week)? Yes

No

Problem 1	Problem 2	Problem 3

Jx4.1. Healthcare sought:

Did you go to a health facility or see a doctor/nurse for this problem? Yes

No

Problem 1	Problem 2	Problem 3

Jx4.1.1. Traditional Healer:

Did you go to a traditional healer, traditional doctor, or bone setter for this problem? Yes

No

Jx5.1. Type of healthcare received: *[Only when 'Yes' to J4.1]*

Problem 1	Problem 2	Problem 3

What kind of treatment did you receive?

None / No surgical care

Major procedure = a procedure which requires regional/general anesthesia

Minor procedures = dressings, wound care, punctures, suturing and I&D

Institutional Review Board
American University of Beirut

10 MAY 2021

APPROVED

Jx6.1. Reason for not having surgical care: *[Only when 'none / no surgical care' to J5.1 or 'no' to J4.1]*
What was the main reason not to go to a health facility to see a doctor/nurse or not to have an operation or dressings?
*[If the person went to a traditional healer ask why (s)he didn't go to a health care facility and mark that as the answer.
If the person was referred but did not go to the referral hospital, mark the answer why (s)he did not go here.]*

Problem 1	Problem 2	Problem 3

No money for health care
No (money for) transportation
No time
Fear / no trust
Not available (facility/personnel/equipment)
No need

Jx7.1. Disability:

Problem 1	Problem 2	Problem 3

Does this problem still impact your daily life?

The condition is not disabling
I feel ashamed
I'm not able to work like I used to
I need help with transportation
I need help with daily living

[Disability: a physical problem that impacts your life, or makes it difficult to carry out your daily activities.]

[ASK IF THE PERSON HAD ANOTHER PROBLEM REGARDING THIS ANATOMICAL LOCATION. IF SO, GO TO THE FOLLOWING ROW, PROBLEM 2 OR 3, TO REGISTER THIS PROBLEM. IF HERE ARE NO OTHER PROBLEMS REGARDING TO THIS LOCATION YOU CAN GO TO THE FOLLOWING ANATOMICAL LOCATION. IN CASE OF MORE THAN 3 PROBLEMS, MARK THE ONES WHICH ARE MOST PRESENT AND MOST RELEVANT FOR THE RESPONDENT]

K. EXTREMITIES

Kx1. Extremities:
Have you ever had an injury, burn, wound, mass, deformity, broken bone, or an operation on your hands, feet, arms, or legs?
___ Yes
___ No
[If there were/are no problems with this anatomical section you can continue with the following. If the person had a problem with this anatomical section you question first on problem 1 all the questions on this page and go further if here are more problems]

Problem 1	Problem 2	Problem 3

Kx2.1. Extremity location:
On what part of your body did the problem occur?

Finger(s)
Thumb / Hand
Lower arm
Upper arm
Foot
Lower leg
Upper leg

Institutional Review Board
American University of Beirut

10 MAY 2021

APPROVED

[If the problem is right and left: make two separate tabs to document the problem.]
[If the problem is based on a joint you need to choose the most proximate location to the abdomen.
For example: an elbow fracture is documented as the upper arm, a knee skin contracture is documented as the upper leg.]

Kx3.1. Extremity specifics:

Problem 1	Problem 2	Problem 3

Tell me what problem you have had.

Wound injury related
Wound not injury related
(Recurrent) drainage / discharge
Burn
Mass / growth
Deformity congenital
Deformity acquired

[Recurrent) drainage / discharge from small sore or opening in the skin: a high suspicion for osteomyelitis, infected bone
Deformity: An abnormal tissue arrangement, malformation
Congenital: The person is born with the problem. Think about: clubfeet
Acquired: The person got the problem later in life. Think about: broken bones and scar contractures]

Institutional Review Board
American University of Beirut

10 MAY 2021

APPROVED

Kx3.1.1 Type of injury / accident:
Did the problem started after an injury or accident? What kind of accident?

Problem 1	Problem 2	Problem 3

[Pick the one that best describes the injury / accident. Pedestrian and bicycle crash definition: there was no motorized vehicle involved. All can be intentional or unintentional.]

No, it was not due to an injury / accident
Car, truck, bus crash
Motorcycle crash
Pedestrian, bicycle crash
Gunshot
Stab / slash / cut / crush
Bite or animal attack
Fall
Open fire / explosion
Hot liquid / hot object

Kx4.1. Timing:

Problem 1	Problem 2	Problem 3

When did this problem start?

In the last month
During the past 12 months but longer than a month ago
Longer than 12 months ago

Problem 1	Problem 2	Problem 3

Kx4.1.1 At this moment:
Do you have this problem now (or during the last week)? Yes
No

Problem 1	Problem 2	Problem 3

Kx5.1. Fracture:
Did you break a bone or dislocate a joint? Yes
No

Problem 1	Problem 2	Problem 3

Kx6.1. Healthcare sought:
Did you go to a health facility or see a doctor/nurse for this problem? Yes
No

Problem 1	Problem 2	Problem 3

Kx6.1.1. Traditional Healer:
Did you go to a traditional healer, traditional doctor, Yes

Institutional Review Board
American University of Beirut

10 MAY 2021

APPROVED

or bone setter for this problem? No

Kx7.1. Type of healthcare received: *[Only when 'Yes' to K6.1]*

What kind of treatment did you receive?

Problem 1	Problem 2	Problem 3

None / No surgical care
Major procedure = a procedure which requires regional/general anesthesia
Minor procedures = dressings, wound care, punctures, suturing and I&D Manipulation / casting / sling
Traction

[Internal / external fixation: the bone is fixed with an operation. Sometimes the metal needs to be removed afterwards.
Manipulation / casting / sling: the broken bone was immobilized for some time.
Traction: the patient needed to stay in a hospital bed for a long time, the broken limb was fixed to the bed or a heavy object.]

Kx8.1. Reason for not having surgical care: *[Only when 'none / no surgical care' to K7.1 or 'no' to K6.1]*

What was the main reason not to go to a health facility to see a doctor/nurse or not to have an operation or dressings?

Problem 1	Problem 2	Problem 3

[If the person went to a traditional healer ask why (s)he didn't go to a health care facility and mark that as the answer.
If the person was referred but did not go to the referral hospital, mark the answer why (s)he did not go here.]

No money for health care
No (money for) transportation
No time
Fear / no trust
Not available (facility/personnel/equipment)
No need

Kx9.1. Disability:

Problem 1	Problem 2	Problem 3

Does this problem still impact your daily life?

The condition is not disabling
I feel ashamed
I'm not able to work like I used to
I need help with transportation
I need help with daily living

[Disability: a physical problem that impacts your life, or makes it difficult to carry out your daily activities.]

[ASK IF THE PERSON HAD ANOTHER PROBLEM REGARDING THIS ANATOMICAL LOCATION. IF SO, GO TO THE FOLLOWING COLLUMN, PROBLEM 2 AND/OR 3, TO REGISTER THIS PROBLEM. IF HERE ARE NO OTHER PROBLEMS REGARDING TO THIS LOCATION YOU CAN GO TO THE FOLLOWING ANATOMICAL LOCATION. IN CASE OF MORE THAN 3 PROBLEMS, MARK THE ONES WHICH ARE MOST PRESENT AND MOST RELEVANT FOR THE RESPONDENT]

Institutional Review Board
American University of Beirut

10 MAY 2021

APPROVED

L. WOMEN'S HEALTH

Lx1. Reproductive age screening:
Have you had a bleeding cycle in the last year?
___ Yes
___ No
___ Girl under the age of 12 years
___ Male

[IF 'NO', YOU CAN SKIP ALL THE FOLLOWING QUESTIONS TILL QUESTION L19. ABOUT FAMILY PLANNING.IF 'MALE' OR 'GIRL UNDER THE AGE OF 12 YEARS' YOU CAN SKIP ALL THE FOLLOWING QUESTIONS AND GO TO THE END OF THIS FORM TO FINISH THE SURVEY WITH A LAST CHECK OF THE SURVEY AND YOUR SIGNATURE]

Lx2. Gravida:
How many times, including the miscarriages and current pregnancy, have you been pregnant?

_____

[If the answer is 0, continue with question L11]

Lx3. Pregnant:
Are you currently pregnant?
___ Yes
___ No
___ I don't know

Lx3.1. Bleeding during pregnancy: *[Only for those who are currently pregnant.]*
Have you had bleeding during your current pregnancy?
___ Yes
___ No

Lx4. Gestational Age: *[Only for those who are currently pregnant.]*
How many months are you pregnant?

_____

Lx5. Parity: ***[THIS CANNOT BE MORE THAN THE ANSWER TO QUESTION L2]***
How many times have you delivered a baby? *[Includes C-sections and instrumental deliveries]*

_____

[If the answer is 0, continue with question L11]

Lx6. Home deliveries: ***[THIS CANNOT BE MORE THAN THE ANSWER TO QUESTION L5]***
How many babies were delivered at home?

_____

Lx7. Health facility deliveries: ***[THIS CAN BE CALCULATED FROM QUESTION L5 MINUS L6]***
How many babies were delivered in a health facility?

_____

Lx8. C-section: ***[THIS CANNOT BE MORE THAN THE ANSWER TO QUESTION L7]***
How many times where your babies delivered with an abdominal delivery / C-section?

_____

Lx9. Instrumental deliveries: ***[THIS CANNOT BE MORE THAN THE ANSWER TO QUESTION L7]***
How many times where your babies delivered with the help of instruments (Vacuum / Forceps)?

_____

Lx10. Breastfeeding:
Are you currently breastfeeding?
___ Yes
___ No

The following questions are about your menstrual period.

Lx11. Length of period:

Institutional Review Board
American University of Beirut

10 MAY 2021

APPROVED

How long does your period last? (number of days)
_____

Lx12. Regularity:
Does your period come regularly?
___ Yes
___ No

Lx13. Intermittent bleeding:
Do you have small bleedings in-between your period?
___ Yes
___ No

Lx14. Pain:
Do you have pain during your period so that you cannot work?
___ Yes
___ No

Lx15. Pads or towels/cloths:
Do you use pads or towels/cloths?
___ Pads
___ Towels/cloths

Lx16. Pads/towels:
How many sanitary pads do you use on the heaviest day of your period?
_____

Lx17. Health care needed:
Is there, in your opinion need for healthcare for your menstrual cycle?
___ Yes
___ No

Lx17.1 Traditional healer:
Is there need to go to a traditional healer for your menstrual cycle?
___ Yes
___ No

Lx18. Possibilities for health care *[only if L17 is Yes]*
Do you have the possibility for health care for your menstrual problem?
___ No, no money for health care
___ No, no (money for) transportation
___ No, no time
___ No, because of fear / no trust
___ No, not available (facility/personnel/equipment)
___ Yes, will go

Lx19. Family Planning:
Do you use a family planning method at the moment? *[Not including traditional methods]*
___ Yes
___ No

Lx20. Type of family planning? *[only if L19 is answered with 'Yes']*
What method do you use currently?
___ Contraceptive pills
___ Implant
___ Injectable
___ Intra uterine device / coil
___ Condom
___ Other (surgical methods)

Thank you very much for answering these questions. Let me check everything.

[Go over all the tabs to ensure that you have everything. Ask the questions again which you accidentally skipped.

I checked the full Survey, there is no data missing:
______________ *[date]* _______________ *[name]* _______________ *[signature of interviewer]*

Institutional Review Board
American University of Beirut

10 MAY 2021

APPROVED

I see that I have everything that I need; your survey is complete. Thanks again on behalf of the research team. Do you have questions for me? You are always welcome to contact us. Have a nice day.

[Give or refer to the letter with the (contact) information about the survey.] [TIME: ____ hour ____ min]

If you wish to make a comment for this interview you can do that below:

____
____
____
____

Institutional Review Board
American University of Beirut

10 MAY 2021

APPROVED
